# Lipid‐Facilitated Opening of the ADAM10 Sheddase Revealed by Enhanced Sampling Simulations

**DOI:** 10.1002/advs.202515713

**Published:** 2026-02-24

**Authors:** Adrien Schahl, Nandan Haloi, Marta Carroni, Shengpan Zhang, Quentin James Sattentau, Erdinc Sezgin, Lucie Delemotte, Rebecca J. Howard

**Affiliations:** ^1^ SciLifeLab Department of Applied Physics KTH Royal Institute of Technology Solna Sweden; ^2^ SciLifeLab Department of Biochemistry and Biophysics Stockholm University Solna Sweden; ^3^ The Kennedy Institute of Rheumatology University of Oxford Oxford UK; ^4^ Sir William Dunn School of Pathology University of Oxford Oxford UK; ^5^ The Max Delbrück Centre for Molecular Medicine Berlin Germany; ^6^ SciLifeLab Department of Women's and Children's Health Karolinska Institutet Solna Sweden

**Keywords:** ADAM10, fluctuation amplification of specific traits, Markov state models, phosphatidylserine, protein‐lipid interactions

## Abstract

ADAM10 is a crucial membrane‐bound metalloprotease that regulates cellular physiology by cleaving and releasing membrane‐anchored proteins, including adhesion molecules and growth factor precursors, thereby modulating cell signaling, adhesion, and migration. Despite its central role, its activation mechanisms are not fully understood. Here, we model how phosphatidylserine (PS) exposure during apoptosis triggers ADAM10 activation. We confirm that PS externalization is associated with ADAM10‐mediated CD43 shedding from the surface of T cells. Intriguingly, ADAM10 activation correlated with loss of ADAM10 monoclonal antibody binding, suggesting a PS‐induced conformational change that alters epitope accessibility. To explore this lipid‐mediated conformational change of ADAM10, we employed molecular dynamics simulations to map its conformational landscape. Our simulations revealed that in the absence of PS, ADAM10 samples predominantly closed and intermediate states. By contrast, the presence of PS destabilizes the closed conformation, thereby favoring open states. We provide a mechanistic explanation for this PS‐induced conformational change, which drives ADAM10 activation and loss of mAb binding through conformational change. These findings offer new insights into the lipid‐mediated regulation of ADAM10 and its conformational dynamics.

## Graphical abstract

Phosphatidylserine acts as a lipid trigger to enhance activation of the sheddase ADAM10. By integrating fluorescence spectroscopy assays with enhanced sampling molecular dynamics simulations, this study shows that phosphatidylserine promotes ADAM10 catalytic activity along with expansion of its extracellular domains, enhancing accessibility to scaffold or substrate proteins. The results shed light on lipid‐dependent dynamics and prospective modulation during apoptosis.

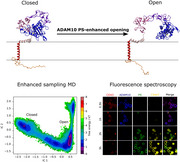

## 1 Introduction

The “A Disintegrin and Metalloproteinase” (ADAM) family of membrane‐anchored enzymes plays essential roles in many biological processes. One of their most important functions is to act as a cell surface sheddase, catalyzing the cleavage and release of multiple substrates including growth and differentiation factors, signaling molecules, and cytokines. Dysregulated ADAM function is associated with a number of diseases including cancer, cardiovascular, neurodegenerative and inflammatory disorders [1]. Given the importance of the correct function of these enzymes, they are tightly regulated at transcriptional, translational and post‐translational levels [2, 3]. Post‐translational regulation includes removal of an auto‐inhibitory pro‐domain during ADAM processing, and complexation with a family of tetraspanins that modulate ADAM specificity and activity [4]. In the case of ADAM10 and 17, it has been recently proposed that phosphatidylserine (PS) flipping to the outer leaflet of the plasma membrane acts as a final trigger for activation [5, 6]. PS is a phospholipid maintained primarily at the inner leaflet of the plasma membrane by flippases [7]. However, under conditions of cell activation and calcium flux, or cell death, PS is relocated to the outer leaflet by the action of scramblases [7]. Thus, scramblase activation likely indirectly modulates ADAM10/17 enzyme activity [8, 9, 10, 11]. It has been hypothesized that the negative charge of PS interacts with a basic motif of amino acids in the membrane‐proximal domain of ADAM17 to activate the enzyme [12]. Mutation of this basic motif inhibits ADAM17 activation [12], as does pharmacological inhibition of the interaction of PS with ADAM members [10, 11, 13]. Whilst this hypothesis is attractive, the structural modifications taking place in the ADAM enzymes leading to PS‐mediated activation have yet to be defined.

Similarly to its close relative ADAM17, ADAM10 cleaves a wide variety of substrates with broad consequences for immune system modulation [14]. Of particular immunological importance, ADAM10 cleaves Notch, FAS‐ligand, LAG3, and TIM3 in T cells, essential for T cell activation and function [14]. Amongst the more recently‐discovered ADAM10 substrates are the transmembrane mucin‐like molecules CD43, MUC‐1, and CD162, which form a major component of the glycocalyx of immune cells including T cells [11, 15]. Apoptotic cells must be rapidly cleared by phagocytes to prevent unwanted inflammation. Induction of apoptosis in T cells activates caspase‐3, which in turn activates the scramblase XKR8 that flips PS to the outer leaflet [16]. This leads to the ADAM10‐mediated shedding of mucins from the T cell surface [11], reducing glycocalyx density and facilitating engulfment by phagocytes [11, 17]. Given ADAM10's role in immune modulation and tissue homeostasis, it is essential to reveal the molecular mechanisms by which PS exposure leads to ADAM10 activation.

**FIGURE 1:**
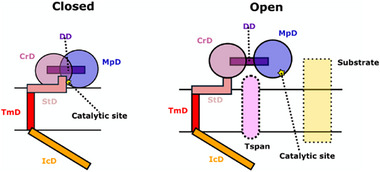
Domains of ADAM10, its interactors and two assumed conformations. The closed model (left) shows tight interactions particularly between the MpD (blue) and CrD (magenta) occluding the catalytic cavity of ADAM10 (yellow star). The open model (right) shows ADAM10 enclosing a Tspan15 protein (pink oval) between the MpD and CrD; the catalytic cavity is turned toward the outside, accessible to cleave substrate (yellow rectangle).

Structurally, ADAM10 comprises seven domains: a pro‐domain, a metallo‐proteinase domain (MpD), a disintegrin‐like domain (DD), a cysteine‐rich domain (CrD), a stalk domain (StD), a transmembrane domain (TmD) consisting of a single membrane‐spanning helix, and an intracellular domain (IcD). Since the prodomain is cleaved upon maturation, only the six remaining domains are found in the plasma membrane (Figure 1).

The MpD is responsible for ADAM10 enzymatic activity, and the CrD appears to be crucial for activation of ADAM10 and its closest homolog ADAM17 [18]. In ADAM17, a small stalk section in the CrD called CANDIS (Conserved ADAM17 Dynamic Interaction Sequence) has been previously shown to form hydrophobic interactions with the membrane, thus triggering CrD structural changes leading to activation. Similarly, in ADAM10, the equivalent region of the protein (here termed StD) has been identified as important for its activation [6]. Particularly, a small patch of cationic residues, namely R657, K659, and K660, positioned in the StD, has been shown to interact with PS, and ADAM10 activity was reduced upon mutation of these residues to asparagine [6].

To date, two distinct structures of ADAM10 extracellular domains have been resolved [19, 20]. The first, obtained through crystallography, depicts a closed‐like conformation where the StD occludes access to the MpD's catalytic cavity. The second, revealed by cryo‐EM, is a complex of ADAM10 with the scaffold protein tetraspanin 15 (Tspan15) and it shows a c‐shaped open conformation exposing the ADAM10 catalytic cavity for substrate access. While this structural opening appears necessary for ADAM10 activity, the structural and dynamic details of this conformational transition, and its modulation by PS, remain to be elucidated.

Molecular dynamics (MD) simulations offer a powerful tool to bridge this knowledge gap by offering atomistic insights into dynamic molecular behaviors [21, 22, 23]. However, large‐scale structural deformations, such as the opening of ADAM10, may require timescales on the order of tens of hundreds of microseconds to observe [24, 25]. Enhanced sampling methods become instrumental in this scenario, enabling the efficient exploration of these conformational transitions [26]. For instance, adaptive sampling methods utilize swarms of short trajectories seeded to target specific transitions between states [27]. By employing Markov state models (MSMs), these trajectories can be stitched together to construct a coherent and comprehensive view of the transition landscape [28, 29].

Here, we experimentally confirm that externalization of PS in apoptotic cells is coordinated with ADAM10 activation by correlating imaging of cell‐surface PS exposure with shedding of a fluorescently labelled ADAM10 substrate (CD43) during apoptosis. This result is consistent with previous analyses linking PS exposure with ADAM10 activation [6, 11]. Surprisingly, ADAM10 activation correlated with the loss of ADAM10 monoclonal antibody (mAb) clone 11G2 binding signal on the cell surface, suggesting a possible conformational change in ADAM10. Based on these observations, we hypothesized that ADAM10 assumes different conformational states with different levels of activity, and that PS engagement triggers a change toward more active states which no longer bind the mAb. To test these hypotheses, we performed MD simulations leveraging the “Fluctuation Amplification of Specific Traits” (FAST) sampling method, yielding trajectories that could be analyzed using MSMs, and thus producing free energy landscapes and transition rates between metastable states. This approach allowed us to characterize the energetic cost of ADAM10 conformational change (here termed “opening”) and to investigate how the presence of PS influences the energetics of this process. Our results reveal that, in the absence of PS lipids, ADAM10 samples a mixture of closed, intermediate and open states, suggesting that activity is possible but sub‐maximal under these conditions. The presence of PS lipids in the outer membrane leaflet, on the other hand, destabilizes the closed state via interactions with the CrD, thereby relatively stabilizing open states compatible with ADAM10 activity. Finally, we show that the presence of PS brings the DD in close proximity to the membrane, which may account for the loss of mAb binding.

## 2 Results

### 2.1 Apoptosis Leads to ADAM10 Substrate Shedding and Loss of ADAM10‐Specific mAb Signal

**FIGURE 2:**
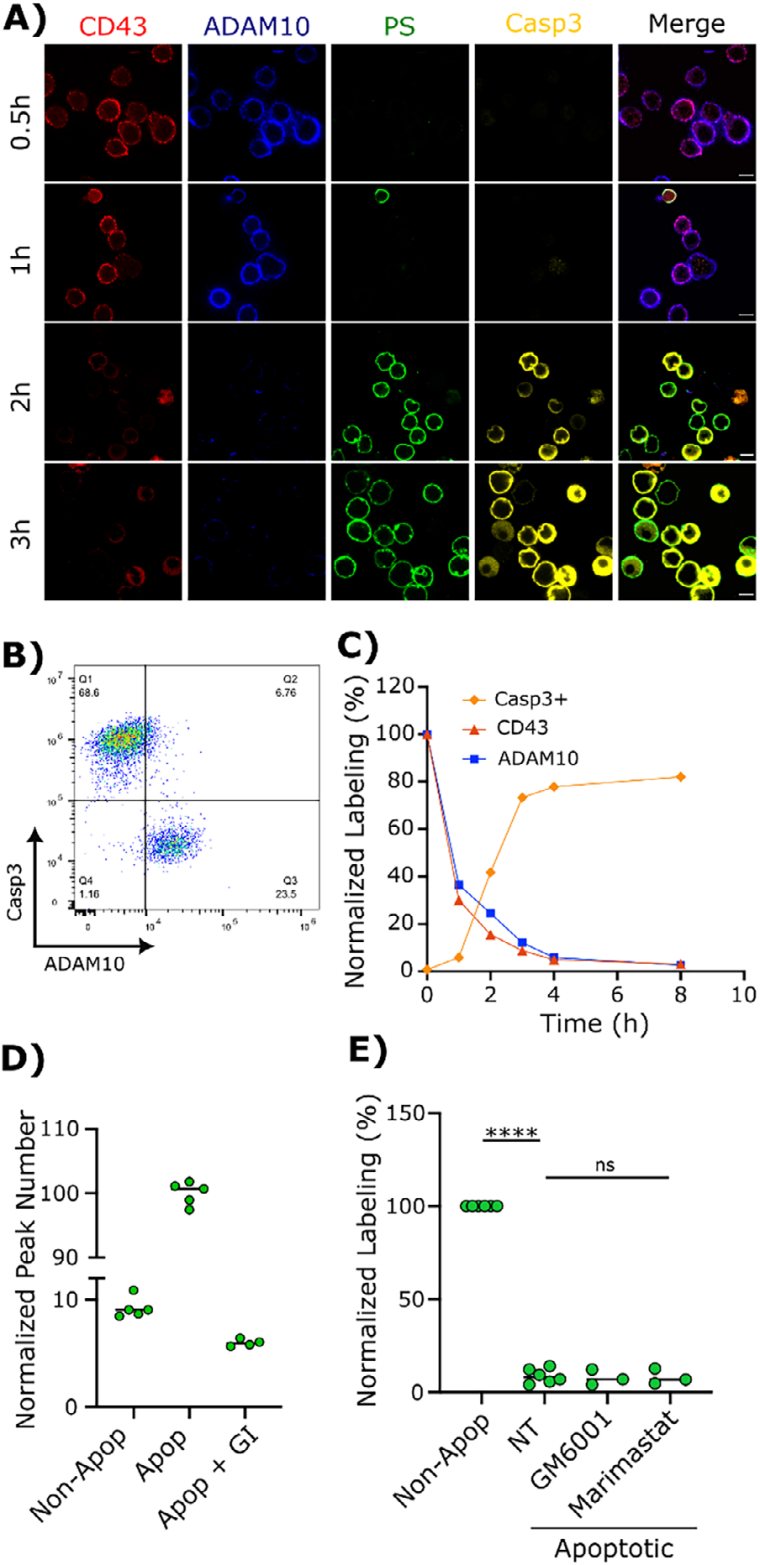
ADAM10 activity upon apoptosis induction. A) The fluorescence signal of anti‐CD43 DFT1 mAb (CD43, red), anti‐ADAM10 11G2 mAb (ADAM10, blue), PS labelled with annexin‐V (PS, green) and activated caspase‐3 (Casp3, yellow) were recorded 0.5, 1, 2, and 3 h after apoptosis induction using fluorescence confocal microscopy. Scale bar = 7 µm. B) Casp3 vs ADAM10 signal analyzed by flow cytometry after 3 h apoptosis induction. C) Casp3 (orange diamonds), ADAM10 (blue squares) and CD43 (red triangles) signal over time quantified by flow cytometry, expressed as % positive normalized to 100% at T = 0 h. D) Molecular quantification of shed CD43Halo in the supernatant normalized to apoptotic cells using single‐particle profiler of cells in the absence (Non‐Apop) and presence of staurosporine‐induced apoptosis (Apop), with and without the specific ADAM10 inhibitor GI. E) ADAM10 signal quantified by flow cytometry in the absence (NT = non‐treated) or presence of broad‐spectrum ADAM sheddase family inhibitors GM6001 or marimastat in Casp3‐negative (Non‐Apop) and Casp3‐positive (Apoptotic) cells, *n* = 3–6 independent experiments, expressed as % positive normalized to 100% ADAM10 expression on non‐apoptotic cells. Solid lines represent mean % ADAM10 expression, with significance tested using one‐way ANOVA of log10‐transformed data with Dunnett's post‐hoc test, ^****^ *p* < 0.0001, ns *p* > 0.05.

To investigate the relationship between PS and ADAM10 activity, we fluorescently labelled ADAM10 and CD43 with mAbs on the human T cell leukemia CEM line. Apoptosis was induced using the kinase inhibitor staurosporine for various times, and confirmed by detection of active caspase‐3 using antibodies, and of externalized PS using fluorochrome‐conjugated annexin‐V. Analysis by confocal microscopy revealed that, as previously described [11], fluorescence intensity of the mucin‐like molecule CD43 decreased over time; unexpectedly, this was coordinated with an equivalent loss of ADAM10 mAb labeling (Figure 2A). By 2 and 3 h post‐staurosporine treatment, all cells that were positive for caspase‐3 and annexin‐V were essentially negative for CD43 and ADAM10 labeling. This result was subsequently confirmed and quantified by flow cytometric analysis of activated caspase‐3 and ADAM10 co‐labeling (Figure 2B), revealing that >90% of cells positive for caspase‐3 were negative for ADAM10. We further quantified caspase‐3, ADAM10 and CD43 signal over time, and revealed a highly correlated and almost complete loss of CD43 and ADAM10 labeling by 4 h post‐apoptosis induction that was negatively correlated with the kinetics of caspase‐3 activation (Figure 2C).

We confirmed that loss of CD43 labeling was due to cleavage, by quantifying shed CD43 extracellular domain from CEM stably transduced with CD43 fused to an N‐terminal Halo tag (CEM‐CD43_Halo_). CEM‐CD43_Halo_ cells were labeled with fluorescent Halo substrate and induced to undergo apoptosis using staurosporine. CD43‐Halo‐ectodomain released into the supernatant after apoptosis was quantified using single‐particle profiler [30] (Figure 2D). At 3 h after apoptosis induction, we observed substantially higher numbers of CD43 soluble events compared to the healthy cell condition. Moreover, when we used a specific ADAM10 inhibitor (GI254023X, here termed GI), the number of soluble CD43 events was reduced dramatically to below the non‐apoptosis condition (Figure 2D).

Since the ADAM10‐specific mAb was directly conjugated to a fluorochrome, the loss of signal over time cannot result from antigen‐antibody internalization, as the mAb signal would have been maintained intracellularly and therefore still give positive signal by flow cytometry. ADAM10 has both autocatalytic activity and is targeted for cleavage by other ADAM proteases [31, 32]. To exclude ADAM10 ectodomain cleavage as a mechanism of loss of antibody signal, we tested broad‐spectrum metalloprotease inhibitors GM6001 and marimastat, which potently inhibit metalloprotease catalytic activity. Figure 2E shows that these inhibitors had no effect on the loss of ADAM10 mAb binding during apoptosis as defined by activated caspase‐3 labeling, strongly suggesting that loss of mAb binding was due to conformational change in ADAM10 rather than its proteolytic cleavage.

These experiments yielded intriguing observations that necessitate further molecular understanding: i) how might PS (and other lipids) interact with ADAM10 to change its conformation? ii) how are the different conformations of ADAM10 linked to its function and antibody binding? In the following sections, we address these questions using MD simulations.

### 2.2 Distribution of ADAM10 Closed and Open Conformations Obtained by Enhanced Sampling Simulations

**FIGURE 3:**
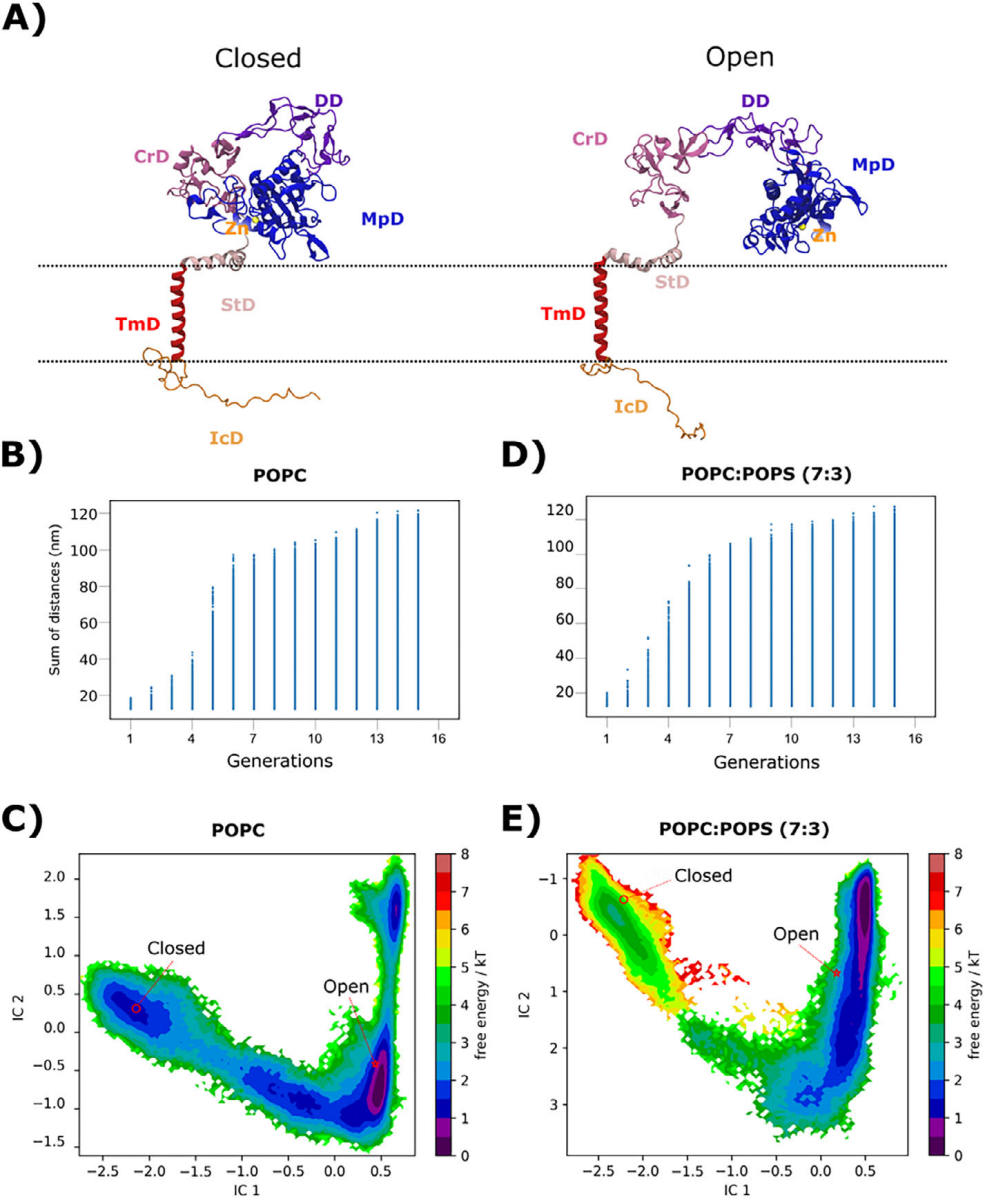
Distribution of ADAM10 closed and open conformations obtained by enhanced sampling simulations. A) Reference models of ADAM10 based on experimental structures in closed (PDB ID: 6BE6, left) and open (PDB ID: 8ESV, right) conformations. Coloring indicates the catalytic zinc ion (yellow), MpD (blue), DD (purple), CrD (magenta), StD (pink), TmD (red) and IcD (orange). B) Scatter plot of summed interdomain distances (Å) used to select seeds for successive generations of FAST sampling in the absence of PS. C) Free‐energy landscape built from an MSM of ADAM10 opening in the absence of PS, plotted on the two slowest tICA components (IC2 vs. IC1) and colored by free energy according to the scale bar (k–1T–1). D) Scatter plot of summed distances as in panel B, used to select seeds for successive FAST generations in the presence of PS. E) Free‐energy landscape as in panel C, built from an MSM of ADAM10 opening in the presence of PS. Landscapes in panels C and E include projections of reference models shown in panel A in closed (red circle) and open (red star).

As we sought to understand the molecular mechanisms responsible for PS‐mediated ADAM10 activation, we first performed extensive MD simulations in a reference membrane environment. Considering available experimental structures, we reasoned that ADAM10 activation would require opening of the protein in order to accommodate both the scaffold (e.g. Tspan15) and substrate. Such a conformational change is expected to take place over hundreds of µs to seconds, far beyond accessible timescales of typical atomistic MD simulations. We therefore decided to tackle this issue using enhanced sampling simulations, specifically the FAST adaptive sampling method. As detailed in Methods, this approach involves running successive unrestrained MD simulations, iteratively using seeds selected based on expected progress toward a target state. The initial model was based on an experimental structure of ADAM10 in an apparent closed conformation (PDB ID: 6BE6) inaccessible to scaffold or substrate proteins, embedded in a homogenous 1‐palmitoyl‐2‐oleoyl‐glycero‐3‐phosphocholine (POPC) bilayer, thus mimicking a non‐activated condition (Figure 3A). We hypothesized that the transition from closed to open conformations, the latter based on a structure resolved in the presence of Tspan (PDB: 8ESV), could be modeled by using as target features the distances between the catalytic MpD and the CrD/StD regions (Figure 3A; Figure S1). We thus defined as a target for FAST sampling (see Methods) maximizing the sum of 127 pairwise Cα distances between these domains (Figure S1 and Table ST1). Over 320 separately seeded simulations of 100 ns each (for a total simulation time of 32 µs), the maximum cumulative sum of interdomain pairwise distances increased from ∼20 nm to ≥100 nm within ∼8 generations; the distance feature increased only incrementally thereafter, and was taken as converged (Figure 3B). Visual inspection of the final frames from all simulations revealed a progressive opening of ADAM10, while retaining the general fold within each domain (Movie S1).

As also detailed in Methods, we performed time‐lagged independent component analysis (tICA) based on interdomain‐distance features (Figure S1 and Tables ST1 and ST2) to characterize the slowest collective motions sampled by ADAM10. We then projected each simulation frame, along with reference structures, on a 2D space defined by the two slowest motions, represented by the top two independent components IC1 and IC2 (Figure 3C). These components appeared to capture a substantial transition between the initial closed and experimental open models (Figure 3C), as defined in more detail below.

### 2.3 Markov State Models Show PS Lipids Favor Opening Transitions

Based on initial Markov state modeling of our POPC‐embedded simulations using 200 micro‐states (Figure S2), reference closed and open models projected to distinct basins in the free‐energy landscape of ADAM10 (Figure 3C). Our central hypothesis was that the interaction of PS with ADAM10 changes the distribution of its conformations in such a landscape. To test this, we built a new simulation system by embedding the closed ADAM10 model in a 7:3 mixture of POPC:POPS lipids, representing an upper bound for outer‐leaflet PS content under apoptic conditions [16]. Based on this membrane composition, we ran another series of MD trajectories using the FAST‐sampling protocol, producing an additional 32 µs of simulations. As also observed in the POPC‐only system, summed interdomain features converged to ≥100 nm within ∼8 generations (Figure 3D), and visual inspection of final frames from all simulations revealed progressive opening of ADAM10 with retention of individual domain folds (Movie S2). Also similar to the POPC system, tICA based on interdomain‐distance features followed by projection of reference models indicated the closed‐to‐open transition in our POPC:POPS system involved displacements along both IC1 and IC2 (Figure 3E). However, a tICA‐based MSM revealed notable differences in the free‐energy landscape in the presence of PS: in particular, the basin corresponding to the initial closed model was disfavored relative to the one containing the experimental open model (Figure 3E).

**FIGURE 4:**
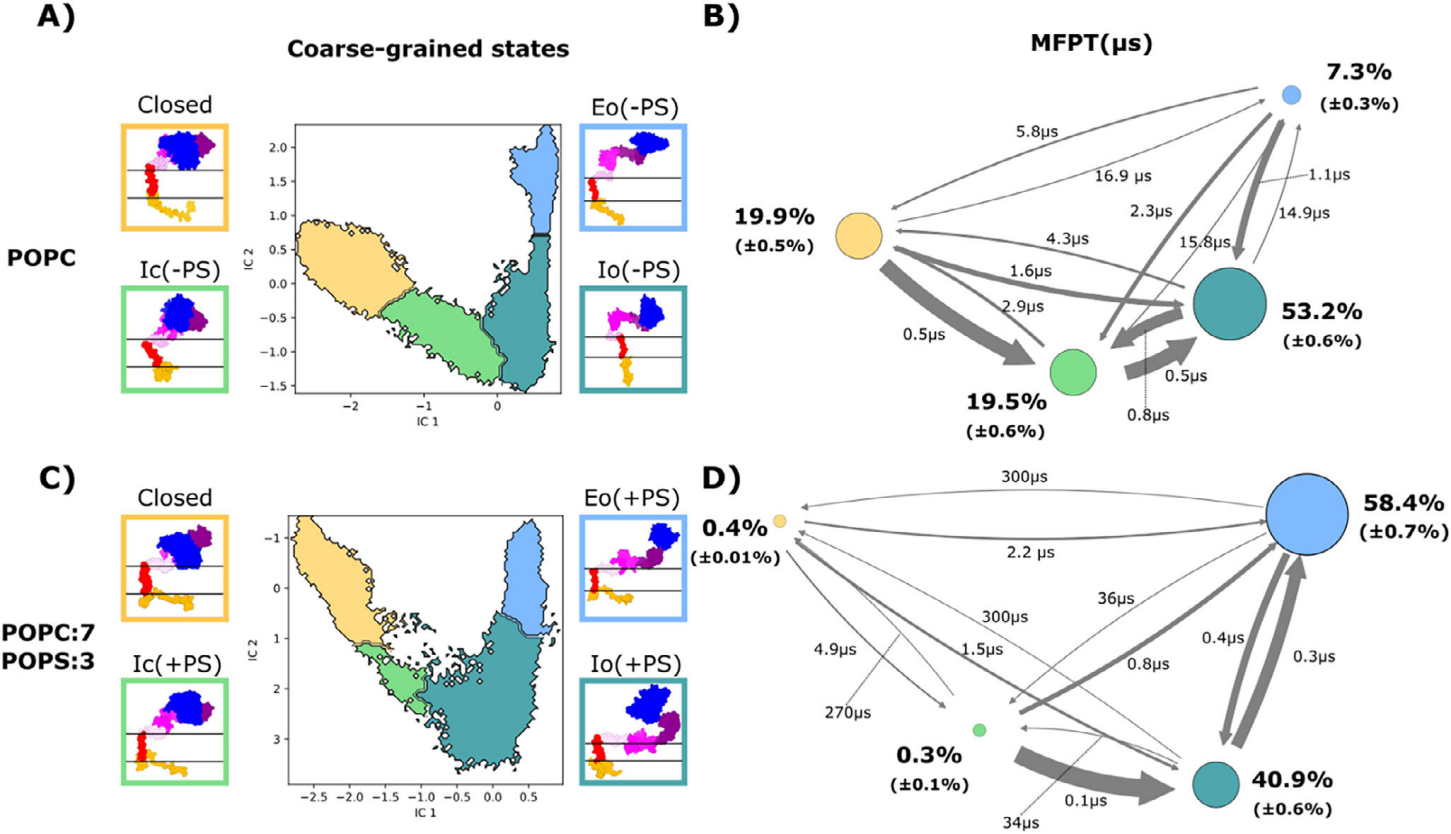
Markov state models show PS lipids favor opening transitions. A) Coarse‐grained MSM computed with PCCA+ based on simulations in POPC alone. Each inset shows a representative simulation frame from the Closed (yellow), IC(‐PS) (green), Io(‐PS) (teal) or Eo(‐PS) (blue) macro‐state, colored accordingly in the IC2‐IC1 plot. B) Kinetic scheme representing exchanges between macro‐states, colored as in panel A, based on simulations in POPC alone. Circles and arrows are scaled according to the relative population in each state and the MFPT between each pair of states, respectively. Errors are based on the Bayesian posterior distribution of each transition, as reported in Table ST3. C) Coarse‐grained MSM as in panel A, based on simulations in 7:3 POPC:POPS alone. Each inset shows a representative simulation frame from the Closed (yellow), IC(+PS) (green), Io(+PS) (teal) or Eo(+PS) (blue) macro‐state, colored accordingly in the IC2‐IC1 plot. D) Kinetic scheme as in panel B, representing exchanges between macro‐states based on simulations in 7:3 POPC:POPS.

To gain more insight into the thermodynamics and kinetics of our ADAM10 conformational landscapes, we then used the PCCA+ algorithm to build a 4‐state coarse‐grained MSM, first for our simulations in POPC alone (Figure 4A). As described in Methods, Bayesian modeling was used to validate the use of a 10‐ns lag time (Figure S3), models were validated using the Chapman‐Kolmogorov test (Figure S4), and errors were estimated for macro‐state populations and mean first‐passage times (MFPTs, the average time that takes to transition from one state to another) following a Bayesian scheme (Table ST3). Based on apparently progressive interdomain expansion, we termed the 4 resulting macro‐states Closed, Intermediate‐closed (Ic(‐PS)), Intermediate‐open (Io(‐PS)) and Expanded Open (Eo(‐PS)) (Figure 4A). In pure POPC, the initial experimental model projected to the Closed state, while the open experimental model projected to the Io(‐PS) state (Figure 3C). Extracellular domains in the Ic(‐PS) state appeared to be partially expanded relative to the initial closed model, while the Eo(‐PS) state was even more expanded than the experimental open model. The Closed and Ic(‐PS) states were present in similar proportions, representing 19.9% and 19.5% respectively; the Io(‐PS) state was the most represented with 53.2% of the total distribution, while the Eo(‐PS) state accounted for the remaining 7.3%. This distribution is consistent with our experimental results, where we showed that ADAM10 presents basal activity (Figure 2E), presumably corresponding to a population in non‐closed states capable of substrate interactions. MFPTs indicated that the Closed, Ic(‐PS) and Io(‐PS) states exchange relatively rapidly, with ≤4.1 µs needed for a given transition; in contrast, transition times to the Eo(‐PS) state were on the order of 20 µs (Figure 4B; Table ST3).

We then used the PCCA+ algorithm to build another 4‐state MSM, this time based on our simulations in 7:3 POPC:POPS (Figure 4C). Projecting simulation frames from each macro‐state onto the previous MSM (Figure S5A), or from the previously identified states onto the new MSM (Figure S5B), revealed that states corresponding to the initial closed model were largely superimposable; we accordingly named these Closed, in the presence as well as absence of PS. Indeed, convergent sampling in this region of conformational space was unsurprising, given the initial models for simulations in the absence and presence of PS were based on the same experimental closed structure (PDB ID: 6BE6). Projections of the other three states were less consistently differentiated in tICA space: a general progression from closed to more‐expanded models could be observed, especially along IC1 in each system, but the corresponding simulation frames did not entirely overlap. For instance, in the presence of PS the open experimental model projected to the boundary between the two most expanded states (Figure 3E). Again, this may be expected given that tICA separation is based on the slowest deformations of the system: if interactions with PS lipids alter the dynamics of ADAM10 opening, they could accordingly alter the separation between macro‐states. To emphasize their parallel structural trends as well as potential system specificity, we therefore named the three relatively expanded macro‐states in our second MSM Ic(+PS), Io(+PS), and Eo(+PS) (Figure 4C).

For this system, the Closed and Ic(+PS) states represented only 0.5% and 0.3% of the total distribution respectively, while the Io(+PS) and Eo(+PS) states accounted for 39.8% and 59.2% respectively (Figure 4D; Table ST3). The Io(+PS) and Eo(+PS) states also exchanged rapidly, with MFPTs <0.5 µs. In the presence of PS, transitions from the Closed/Ic‐type states to the Io‐/Eo‐type states were accelerated (MFPTs as low as 0.1 µs), while transitions back to the Closed/Ic‐type states were slower (MFPTs up to 300 µs). These models were thus consistent with the relative destabilization of more‐contracted states in the presence of PS.

### 2.4 PS Limits CrD‐Membrane Dissociation

**FIGURE 5:**
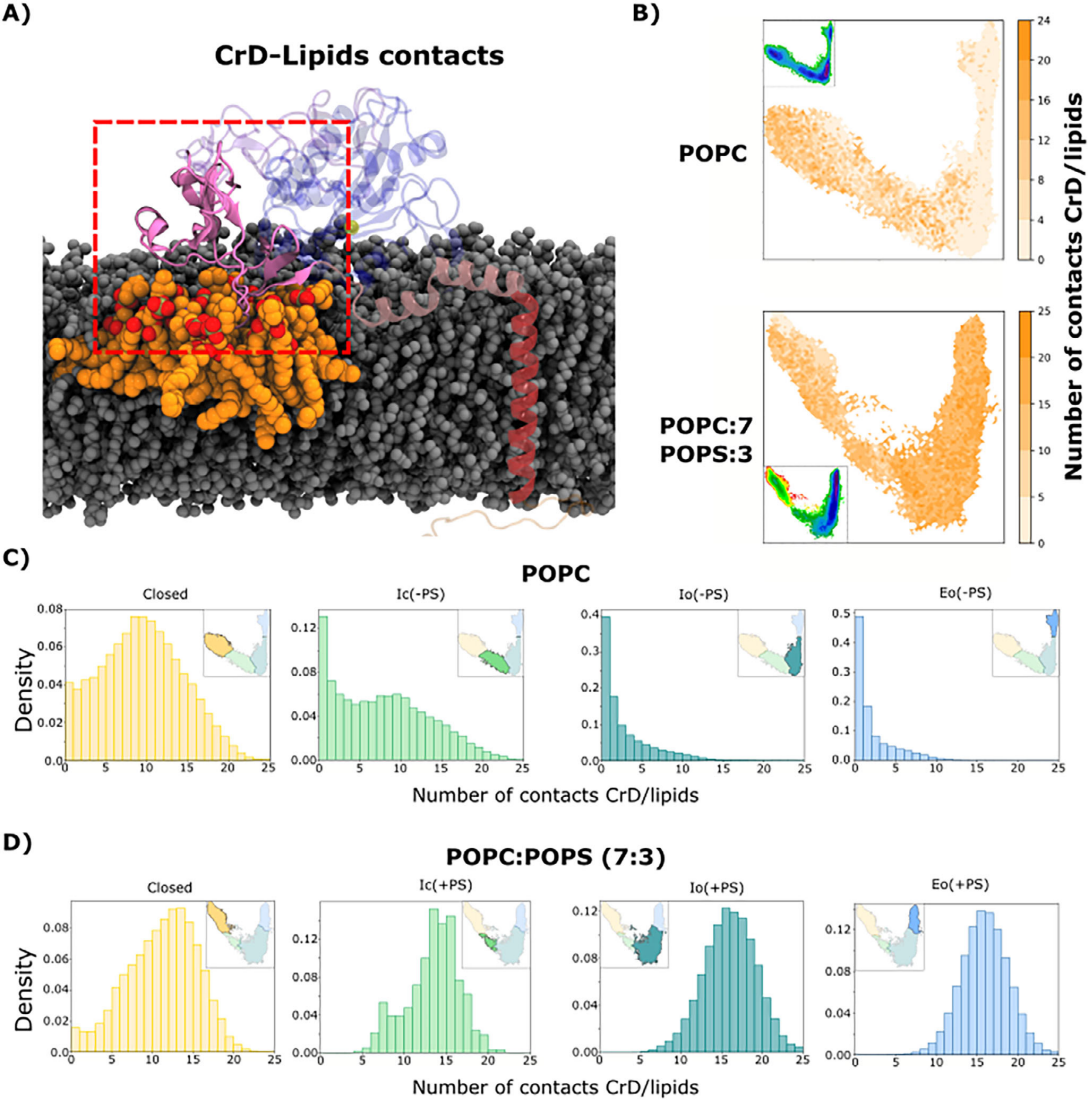
PS limits CrD‐membrane dissociation upon ADAM10 opening. A) Cartoon representation of lipids involved in contacts with the CrD. B) Number of lipid contacts with the CrD in a given simulation frame, colored according to righthand scalebar, projected onto tICA maps based on POPC (above) and POPC‐POPS (below) systems. For reference, insets reproduce free‐energy landscapes from Figure 3. C) Histograms of the number of lipid contacts with the CrD in simulation frames corresponding to the (left to right) Closed (yellow), Ic(‐PS) (green), Io(‐PS) teal or Eo(‐PS) macro‐states obtained in POPC‐only conditions. Insets represent projections of the corresponding macro‐state in tICA space, as originally shown in Figure 4. D) Histograms as in panel C for the (left to right) Closed (yellow), Ic(+PS) (green), Io(+PS) teal or Eo(+PS) macro‐states obtained in 7:3 POPC:POPS.

To understand the molecular details of PS‐dependent sheddase opening, we next quantified contacts between membrane lipids and the membrane‐proximal extracellular domains of ADAM10 in its various states. In the system with POPC alone, the CrD made lipid interactions—defined by any lipid‐protein atom pair within 4 Å—in both the Closed and Ic(‐PS) states, with a distribution centered around 10 direct contacts. However, the distribution of CrD‐lipid contacts peaked at zero in the Io(‐PS) and Eo(‐PS) states, suggesting that POPC lipids are unable to keep the CrD anchored to the membrane during ADAM10 opening (Figure 5A–C).

In contrast, the CrD maintained ∼15 lipid interactions across all states in the 7:3 POPC:POPS system (Figure 5B,D), ∼6 of them specifically with PS lipids (Figure S6A). Residues throughout this domain made occasional lipid contacts (Figure S6B), though a handful of positions—particularly R622, F642 and R646—made 1–3 lipid contacts in at least 30% of simulation frames, across all macro‐states (Figure S6C). Thus, PS interactions—at least in part with specific, bulky residues in the CrD—appeared to prevent dissociation of this domain from the membrane during ADAM10 opening. As expected, these persistent CrD contacts were associated with a lower position of the CrD with respect to the center of the POPC‐POPS membrane, compared to the pure‐POPC condition, particularly in Io and Eo states (Figure S7). Interestingly, lower positioning with respect to the membrane center was also observed for the DD—immediately prior to the CrD in sequence—in the presence of PS in Io and Eo states (Figure S8); membrane proximity of the DD in the POPC‐POPS may be related to mAb insensitivity, as detailed in the last subsection below. The MpD also contacted the membrane in the Closed state (Figure 3A), but unlike the CrD it lost most lipid interactions in the three non‐closed states, both in the absence and presence of PS (Figure S9). Thus, the MpD appears to be consistently solvated relatively early in the opening transition.

**FIGURE 6:**
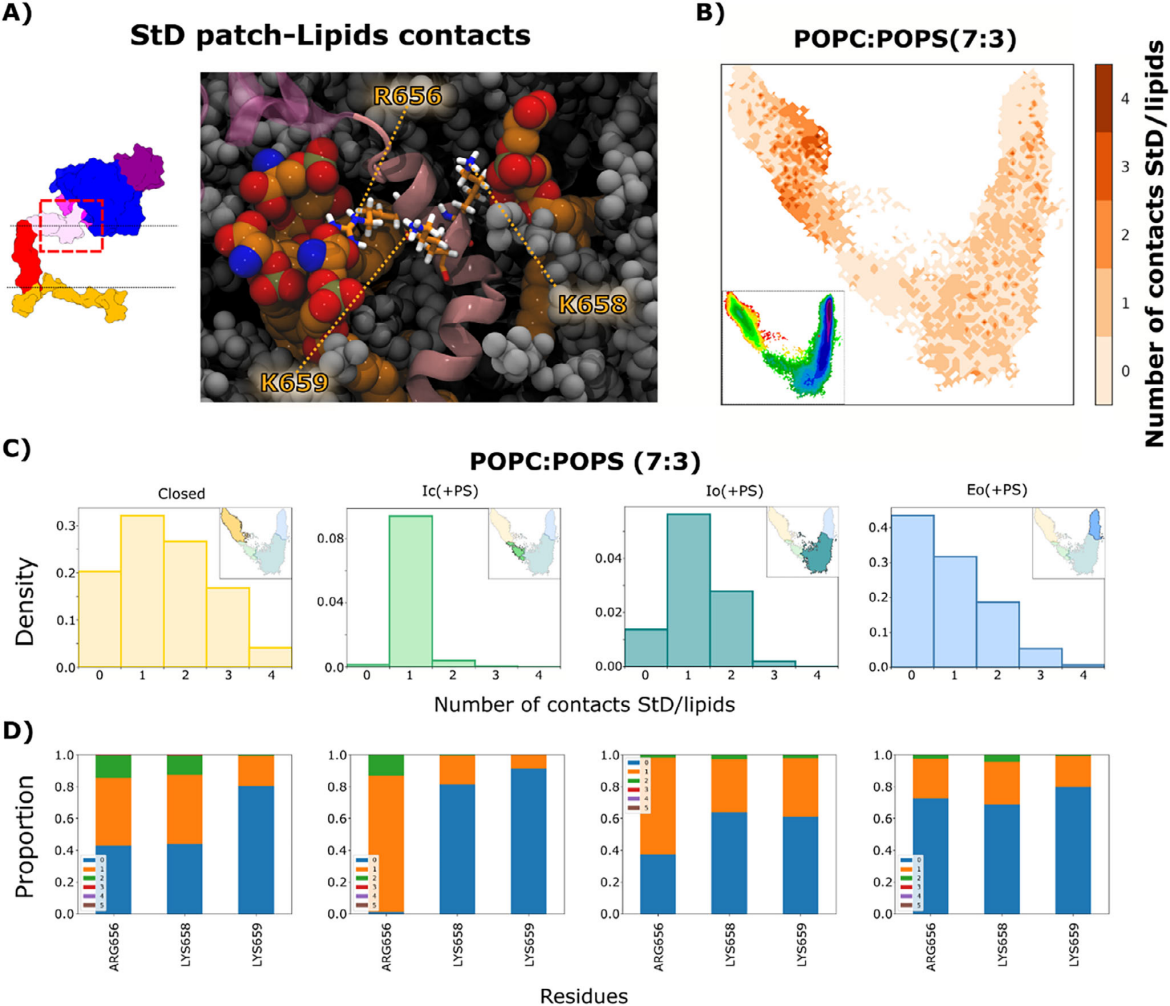
PS interactions with basic residues in the ADAM10 StD. A) Cartoon representation of a cluster of basic residues in the StD (pink) interacting with POPS lipids (orange). Inset shows a schematic of ADAM10, depicted as in Figure 4, with dashed red box indicating the zoomed StD region shown in main panel. B) Number of PS contacts with the StD in a given simulation frame, colored according to righthand scalebar, projected onto a tICA map based on the POPC‐POPS system. For reference, inset reproduces the POPC‐POPS free‐energy landscape from Figure 3E. C) Histograms of the number of PS contacts with the StD in simulation frames corresponding to the (left to right) Closed (yellow), Ic(+PS) (green), Io(+PS) teal or Eo(+PS) macro‐states obtained in POPC‐POPS conditions. Insets represent projections of the corresponding macro‐state in tICA space, as originally shown in Figure 4C. D) Proportion of simulation frames in which a given residue in the StD‐basic patch makes 0 (blue), 1 (orange), 2 (green) etc. contacts with PS lipids in each macro‐state defined in panel C.

A conserved cluster of basic residues (R656, K658, K659) within the StD (Figure 6A) had previously been implicated in promoting ADAM17 activity [12, 33]. We therefore also evaluated the number of contacts between PS lipids and these basic residues in ADAM10. For this domain, the largest number of contacts was apparent in the Closed state, primarily involving R656 (Figure 6B–D). Thus, this patch could help recruit PS lipids in a region proximal to the CrD prior to sheddase opening, though it appears unlikely to mediate direct lipid contacts in relatively expanded states.

### 2.5 PS‐Facilitated Opening Enables Access to Tspan and the Catalytic Site

**FIGURE 7:**
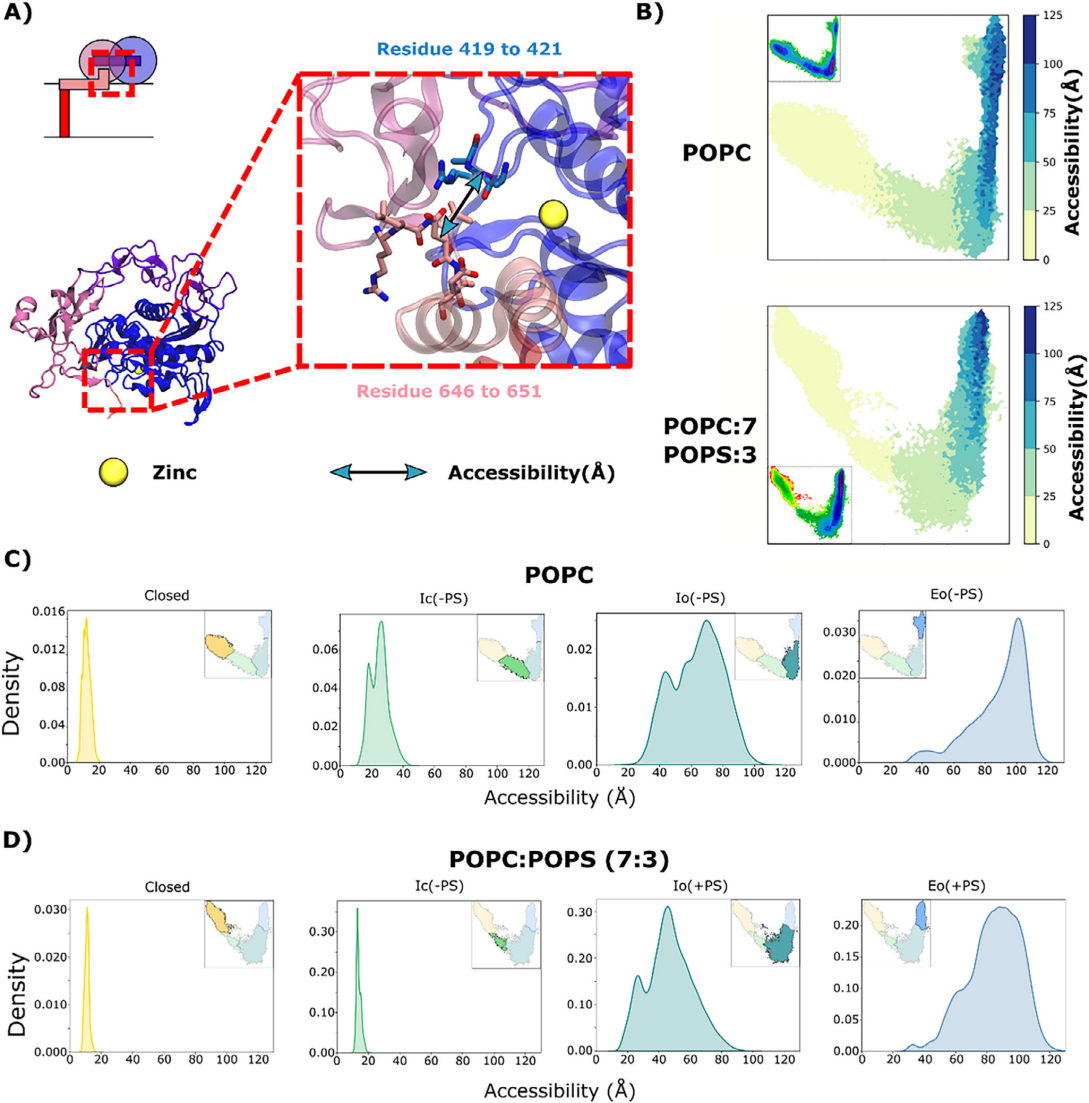
PS‐facilitated opening enables access to Tspan and the catalytic site. A) Schematic (top) and cartoon (bottom) of ADAM10 extracellular domains. Inset shows zoom view of residue clusters on the MpD (blue) and StD (pink) defining the interdomain distance used here to quantify accessibility to the catalytic site (yellow). B) Interdomain distances illustrated in panel A, colored according to righthand scalebar, projected onto tICA maps based on the POPC‐only (above) or POPC‐POPS (below). For reference, insets reproduce free‐energy landscapes from Figure 3. C) Probability distributions of the interdomain distance illustrated in panel A for each macro‐state obtained in the (left to right) Closed (yellow), Ic(‐PS) (green), Io(‐PS) teal or Eo(‐PS) macro‐states obtained in POPC‐only conditions. Insets represent projections of the corresponding macro‐state in tICA space, as originally shown in Figure 4. D) Distributions as in panel C for the (left to right) Closed (yellow), Ic(+PS) (green), Io(+PS) teal or Eo(+PS) macro‐states obtained in 7:3 POPC:POPS.

Having characterized a generalized opening transition of ADAM10 presumed to precede scaffold or substrate binding, we further investigated the accessibility of the functionally critical catalytic site across the same free‐energy landscapes. We first quantified catalytic‐site accessibility on the basis of the center‐of‐mass distance between MpD residues 419–421 (surrounding the catalytic zinc ion) and StD residues 646–651 (Figure 7A), largely represented by variations along IC1. Accessibility increased from ≤20 Å to 25–30 Å between Closed and Ic(‐PS) states in pure POPC, while staying around 20 Å in POPC:POPS conditions. In the absence of PS, the Io(‐PS) state was characterized by a range of accessibility values centered around 65 Å, while the Eo(‐PS) state was relatively expanded to around 100 Å (Figure 7B,C). Similarly in the presence of PS, accessibility in the Io(+PS) state was centered around 50 Å, while in the Eo(+PS) state it was broadly distributed between 50 and 100 Å (Figure 7B,D). Thus, the catalytic site appeared to be similarly accessible in the Io and Eo states in both systems; PS‐dependent differences in activity may be influenced by the relative favorability of these states, more than by access to the catalytic site itself. Accessibility of the Tspan binding cleft was similarly increased in relatively open versus closed states, both in the absence and presence of PS (Figure S10, residues defined in Table ST2). Thus, progressive opening of ADAM10 promotes access to Tspan binding as well as the catalytic site, though a link to PS facilitation was not readily apparent.

### 2.6 PS Is Predicted to Disrupt mAb Binding to ADAM10 by Reducing DD‐Membrane Distance

**FIGURE 8:**
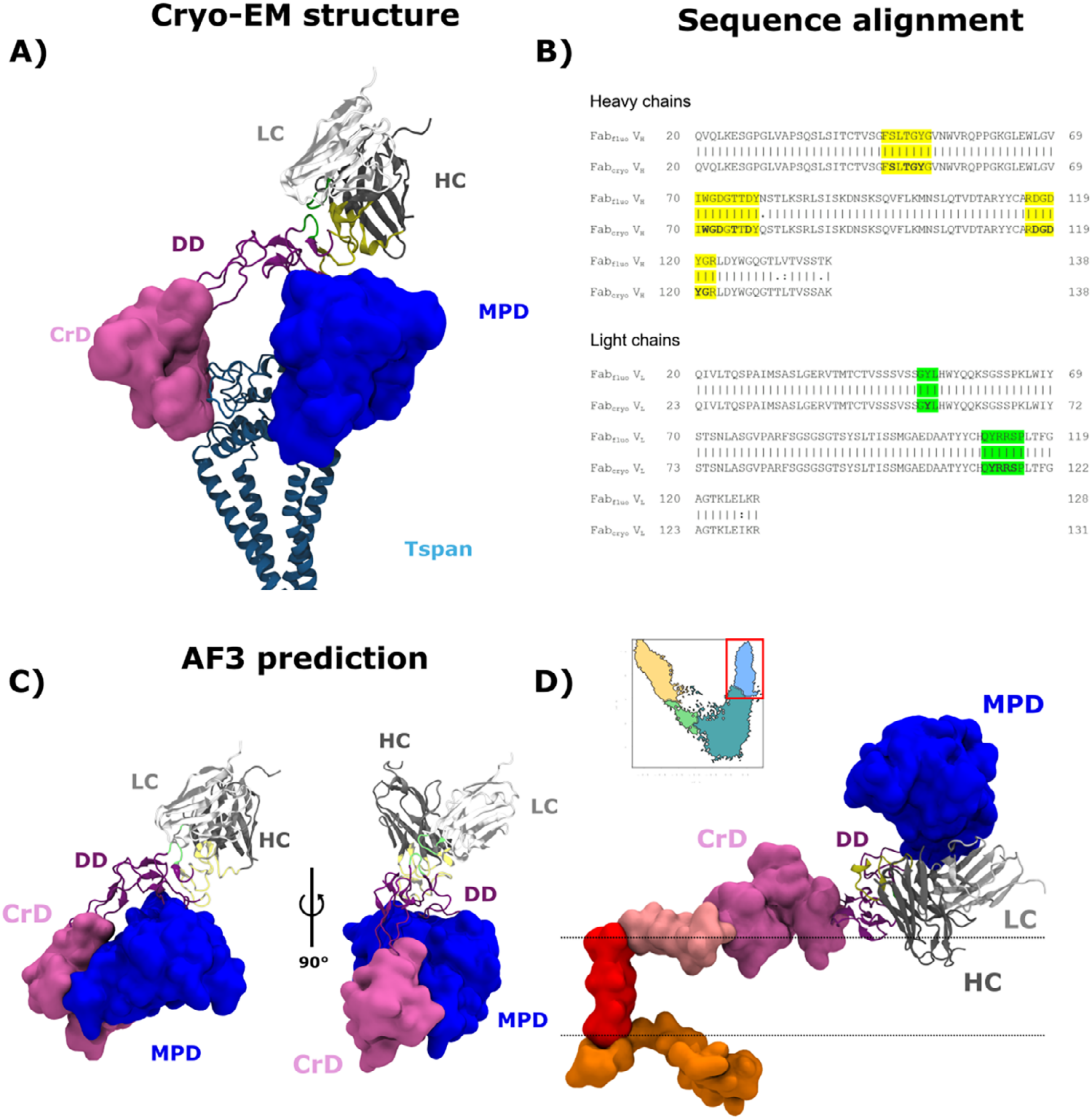
Conformational modulation of ADAM10 interaction with mAb. A) Representation of the Fab_cryo_‐ADAM10 complex determined by cryo‐EM (PDB ID: 8ESV), showing detailed interactions of the Fab_cryo_ heavy chain (HC, yellow) and light chain (LC, green) with the ADAM10 DD (purple). In the experimental structure, the DD is located on the extracellular side of the CrD (pink), MpD (blue), and membrane‐embedded Tspan (teal), avoiding any clash between Fab_cryo_ and the membrane. B) Sequence alignment between Fab chains used in our study (Fab_fluo_, above) and in the cryo‐EM structure in panel A (Fab_cryo_, below). Contacts of the heavy and light chains with the ADAM10 DD in the experimental structure are highlighted yellow and green, respectively. C) Representative model of the Fab_fluo_‐ADAM10 complex generated by AlphaFold3, colored and labeled as in panel A (additional models in Movie S3). D) Predicted pose of Fab fragments, based on superimposition of the experimental‐structure DD, in a representative simulation frame corresponding to the Eo(+PS) state (the one most frequently sampled in the POPC‐POPS condition). Due to rearrangement of the DD in simulations with PS, portions of the Fab clash with the predicted membrane region (horizontal lines).

Having defined effects of PS on the ADAM10 conformational landscape, we sought to test whether PS‐induced structural changes might contribute to the loss of ADAM10‐mAb binding we observed in our fluorescence experiments (Figure 2). We determined that the Fab heavy chain used in our experiments (Fab_fluo_) has an identical antigen‐binding sequence as the one in the ADAM10 cryo‐EM structure (Fab_cryo_ in PDB ID: 8ESV, Figure 8A,B), used here as a representative open state. In the experimental structure, Fab_cryo_ binds to the ADAM10 DD (Figure 8A). We also generated models of the ADAM10‐Fab_fluo_ complex using the AlphaFold3 server (https://alphafoldserver.com), and observed similar interactions with the ADAM10 DD (Figure 8C, Movie S3). In the experimental structure, Fab_cryo_ heavy‐chain residues 48–54, 71–77, and 118–123 directly contact ADAM10‐DD residues 475–498, while Fab_cryo_ light‐chain residues 113–118 contact ADAM10‐DD residues 491–500 (Figure 8A,B). In AlphaFold3 predictions, Fab_fluo_ heavy‐chain residues 48–51 and 71–77 contact ADAM10‐DD residues 473–493, while light‐chain residues 51–53 and 110–115 contact ADAM10‐DD residues 491–500 (Figure 8B,C), defining substantially conserved interactions. As described above, the DD is positioned closer to the membrane in Io/Eo states in the presence versus absence of PS (Figure S8); therefore, we hypothesized that Fab binding to the DD might be occluded by membrane proximity in PS simulations. In closed states, and generally in POPC‐only conditions, the DD was farther from the membrane (Figure S8) and could be more accessible to Fab binding.

To test this hypothesis, we randomly extracted 10 simulation frames from the most populated state (Eo(+PS)) in the POPC‐POPS system. Since our sequence alignments and AlphaFold3 predictions indicated Fab_fluo_ interacts with ADAM10 in a similar manner as Fab_cryo_, we approximated the Fab_fluo_ pose by superimposing the experimentally determined Fab_cryo_ complex onto each extracted frame, based on the ADAM10 DD. Indeed, for 9 out of 10 frames, the pose of Fab_cryo_ with respect to ADAM10 clashed with the membrane (Figure 8D; Figure S11). Thus, conformational changes in ADAM10 during simulations with PS, particularly reorienting the DD with respect to the membrane, likely contribute to disruption of antibody binding.

## 3 Discussion

Structural and dynamical characterization of ADAM10 is crucial for understanding its function. Similar to other enzymes, its activity is closely linked to its propensity to adopt different conformational states and interconvert between them. Gaining insight into how ADAM10 transitions between different conformations can provide valuable information about its regulation and activation mechanisms, which are key to its biological roles.

Until recently, only a closed structure of ADAM10 had been defined [19]. However, a more recently published structure revealed an open and active conformation, enclosing a Tspan15 protein [20]. It thus appeared that the opening of ADAM10 was an important step toward its ability to cleave membrane substrates. As we and other groups previously showed that the presence of PS in the outer membrane can enhance ADAM10 activity [6, 11], we hypothesized that the opening of ADAM10 would be modulated by the presence of these lipids in the plasma membrane. Our MSM analysis shows that, in a pure POPC membrane, closed and open experimental structures project into two distinct metastable basins, with the open conformation appearing to be slightly more stable than the closed one. When analyzing the behavior of ADAM10 in a 7:3 POPC:POPS membrane mixture, we observed striking differences in its conformational dynamics compared to a pure POPC membrane. Most notably, the closed structural basin was severely destabilized relative to the open one. These results indicate that PS enhances opening of ADAM10 as well as its functional activity.

To quantify PS‐dependent shifts in the ADAM10 conformational landscape, we further constructed a coarse‐grained MSM comprising four states, ranging from the most closed to the most open: Closed, Ic(‐PS), Io(‐PS), and Eo(‐PS). Interestingly, while the experimental closed structure aligns with the Closed state, the experimental open structure corresponds to the Io state. This suggests that ADAM10 adopts even more extended conformations in solution than observed thus far by structural methods. The Io(‐PS) state accounts for 52% of simulation frames, making it the most prevalent conformation in pure POPC. This observation sheds new light on ADAM10 structure and dynamics, as it was thought to be mainly in the closed state at the surface of the cell [19, 34], potentially in dimer form. After obtaining the closed structure, Seegar and co‐authors suggested that ADAM10 could undergo transient opening leading to its activation. Here, our models support the concept that ADAM10 can indeed adopt an open conformation in the membrane, without requiring an external stimulus. Our data also confirm basal activity of ADAM10 that can be reduced by the addition of an inhibitor.

**FIGURE 9:**
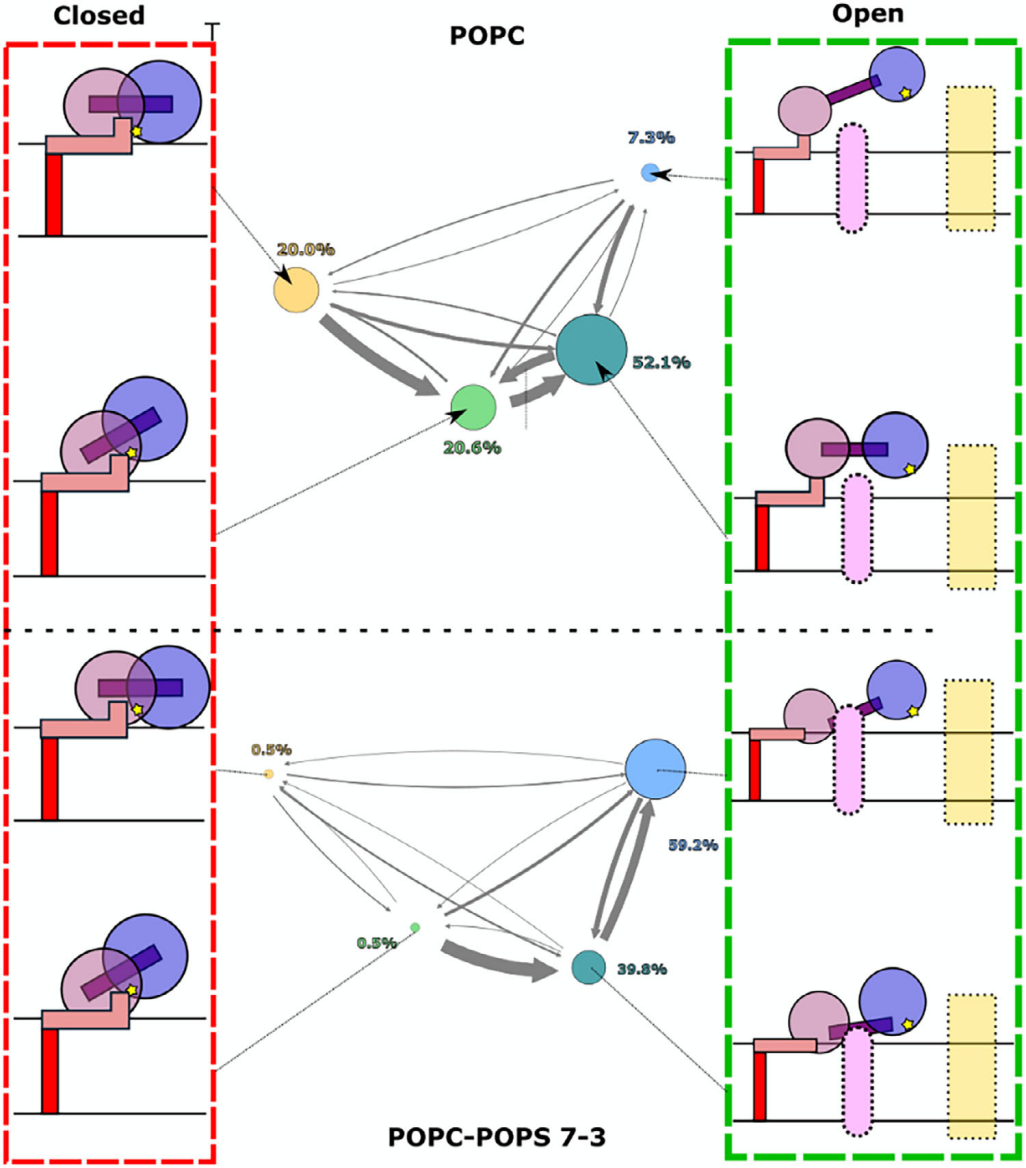
Schematic of conformational transitions in ADAM10 opening in the absence or presence of PS. Without PS (above), ADAM10 oscillates between roughly equivalent proportions of closed (left) and open (right) states. In closed states, the catalytic site (yellow star) is not accessible to substrate, and binding to proteins in the Tspan family is unlikely. Upon addition of PS (below), closed states are disfavored, allowing ADAM10 to bind Tspan (pink oval) or cleave diverse substrates (yellow rectangle).

Another dimension of ADAM10's regulation regards its interactions with Tspan proteins, as observed biochemically [35] and in crystallographic [36] and cryo‐EM structures [20]. Proteins from the Tspan family are known regulators of ADAM10, especially regarding its surface expression [37]. In this context, Tspan15 upregulates the expression of ADAM10 and limits its endocytosis [35]. However, the process of assembly between ADAM10 and Tspan proteins remains an open question, especially since a closed, autoinhibited structure of ADAM10 has been crystallized. Our results suggest that the complex can form even in the absence of PS, e.g. in the homeostatic plasma membrane. Here we note that the open population (∼50%) in our MSM does not explain quantitatively the reduction of activity that we measure in our experiments. As Tspans modulate the selectivity of cleavage of ADAM10, a possible explanation could lie in the competition between different Tspans, as shown previously [37, 38]. The percentage of substrate cleaved in our experiment would then correspond to a subset of the open frames that we observe in our simulations. In this context, fast switching between closed and open states could not only allow ADAM10 to present a basal activity, but also facilitate its binding of Tspan and/or access to substrate (Figure 9).

Upon the addition of PS lipids in our model, we observed a drastic increase in expanded populations, with the Io(+PS) and Eo(+PS) states accounting now for 99% of the sampled frames. Open, accesible forms dominate while closed forms become almost non‐existent, suggesting a structural basis for the increased activity observed with increasing PS. In their work [19], Seegar and co‐authors studied the binding interface of ADAM10 with mAb clone 8C7, which has an enhancing effect on ADAM10 activity. They demonstrated that in the autoinhibitory form, the MpD of ADAM10 would clash with the heavy chain of 8C7, explaining why this mAb engages and stabilizes the active form of the sheddase. This observation, added to our results, highlights the correlation between ADAM10 opening and catalytic activity, and the tight relation between its structure and function.

A key factor in ADAM10 modulation by PS lipids is their interaction with the CrD. These persistent interactions with the membrane could explain why it is difficult for the system to revert from the Io(+PS) state to the Ic(+PS) state, as the CrD remains anchored and restricts conformational reversibility. This ADAM10 behavior is comparable to that of ADAM17, which is modulated by changes in CrD conformation mediated by interactions with negatively charged lipids [21, 39, 40], suggesting a common feature in this family of proteins. Surprisingly, the previously characterized cationic patch present in ADAM17 (R656, K658, K659) displayed increased interactions with PS lipids specifically in the Closed state. These residues may help recruit lipids to ADAM10 prior to opening, which could contribute to subsequent anchoring the CrD and stabilizing its association with the membrane. PS‐mediated repositioning of the CrD also influences the position of the DD, bringing it closer to the membrane in open states. In this pose, the binding of 11G2 Fab would become unlikely due to steric clashes with the membrane, thus explaining the loss of ADAM10 signal that we observed in our experiments. On the other hand, overall opening of ADAM10—characterized by accessibility of the catalytic cavity (Figure 7)—was not notably enhanced by the presence of PS, indicating that the states sampled in the POPC‐POPS membrane retain a similar structural framework to those identified in pure POPC conditions. Together, these findings highlight that the presence of PS not only shifts the equilibrium toward open conformations but also influences specific membrane‐protein interactions that are likely crucial for the activation and function of ADAM10.

A discrepancy remains between the substantial open population observed in both the absence and presence of PS, and the PS‐dependent increase in activity observed experimentally. We envision multiple hypotheses regarding this discrepancy. 1) Following standard reductionist practice in our field, we used simple membrane models, and cannot exclude that alternative lipids such as cholesterol or inner‐leaflet PS could influence the balance between states. As previous work on ADAM10 has focused on the interactions between negatively charged lipids and extra‐cytoplasmic domains of ADAM10, we also did not consider potential PS interactions with ADAM10 at the internal leaflet of the membrane. Sampling errors associated with such simplifications may limit the interpretability of our results. 2) Projection of the Eo(‐PS) state onto the Eo(+PS) state, the most populated in POPC‐POPS conditions, suggests that this state could constitute a critical step toward ADAM10 activation. The increase in Eo population from 7.3% to 58.4% in the presence of PS further correlates with the observed increase in activity of ADAM10. Thus, although the open experimental structure projects closer to Io states, ADAM10 may need to sample an Eo state to accommodate substrates or Tspan scaffolds. 3) Retention of the CrD and MpD near the membrane in the presence of PS may facilitate additional activity‐promoting interactions such as with Tspans, suggesting a dual role for PS in modulating activity as well as substrate accessibility. These hypotheses merit further testing to understand the full molecular mechanism of ADAM10 activation.

In conclusion, the presence of PS shifts ADAM10 toward more open conformations, coordinated with the enhancement of its activity. Understanding how ADAM10 conformational states influence catalysis and specificity could have critical applications in designing agents to either enhance or inhibit its activity, with direct therapeutic relevance.

## 4 Methods

### 4.1 Flow Cytometric Analysis of Antibody Binding to ADAM10 on Healthy and Apoptotic T Cells

Acute T cell lymphoid leukemia (T‐ALL) line CEM was obtained from the Sir William Dunn School of Pathology cell bank. CEM was grown in complete RPMI (ThermoFisher 11875093) containing 10% FCS (Sigma–Aldrich F9665), 1% penicillin/streptomycin (ThermoFisher 15140122) at 37°C/5% CO_2_. CEM was counted and resuspended at a concentration of 2–3 × 10^6^ cells/mL in complete RPMI. Staurosporine (Cambridge Bioscience S‐7600) was pre‐titrated to 10 µm giving ∼50% apoptosis at 3 h post‐treatment. Cells were washed with PBS and collected for further manipulation. Cells were centrifuged for 5 min at 400 x g. Pellets were resuspended in 100 µL cold annexin‐V binding buffer (BD Pharmingen 556454) for 20 min at 4°C in the dark with annexin V‐FITC (Biolegend 640906) used at 1:100, near‐IR fixable viability dye (Invitrogen L10119) at 1:1000, fluorophore‐conjugated anti‐human CD43 sialic acid‐independent clone L10‐APC (Invitrogen 10746863) used at 2 µg/mL and anti‐human ADAM10‐BV421 clone 11G2 (BD Biosciences 742787) used at 2 µg/mL. All labeling was done in conjunction with the corresponding concentration‐matched isotype control antibody. After labeling, cells were washed with cold annexin V binding buffer where appropriate, centrifuged for 2 min at 400 x g at 4°C, and fixed with 4% paraformaldehyde (Sigma‐Aldrich 158127) for 10 min at RT. Fixed cells were washed with PBS, permeabilized with perm buffer (Biolegend 421002), and labeled with anti‐human active caspase‐3 antibody clone Asp175 (Cell Signaling Technologies 9661) used at 1:400. After 30 min incubation at 4°C, cells were washed with FACS wash buffer (PBS, 2% FCS) and incubated with anti‐Rabbit IgG (H+ L) Alexa Fluor 546 (Invitrogen, A11010) used at 4 µg/mL (1:500) where required for 30 min at 4°C. After washing, cells were analyzed by flow cytometry using a Cytoflex LX flow cytometer (Beckman Coulter) and data processed using the FlowJo‐V10 software (FlowJo, LLC). Isotype controls and Fluorescence Minus One (FMO) controls were performed for all colors to gate on positive and negative populations. Gating on the relevant cell population was set according to Forward Scatter (FSC) and Side Scatter (SSC) before doublet and near‐IR fixable viability dye (Thermo Fisher) to allow exclusion of dead cells with dye‐permeable membranes. Subsequent gating for all samples was carried out on annexin V and/or activated caspase‐3 labeling to differentiate apoptotic from non‐apoptotic cells within the same total cell population. Broad spectrum metalloprotease‐specific inhibitors (GM6001, R&D Systems 2983; Marimastat, R&D Systems 2631) were each added at the pre‐determined optimum concentration of 10 µM for 40 min prior to the addition of staurosporine, and maintained during the experiment.

### 4.2 Confocal Microscopy of Healthy and Apoptotic T Cells

CEM were counted and apoptosis induced as for flow cytometric analysis. Cells were washed with PBS resuspended in 100 µL cold annexin‐V binding buffer (BD Pharmingen 556454) for 20 min at 4°C in the dark with annexin V‐FITC (Biolegend 640906) used at 1:100, Alexa Fluor 647 conjugated anti‐human CD43 sialic acid‐dependent clone DFT1 (Santa Cruz Biotechnology, sc‐6256) used at 2 µg/mL and anti‐human ADAM10‐BV421 clone 11G2 (BD Biosciences 742787) used at 2 µg/mL. After labeling, cells were washed with cold annexin V binding buffer where appropriate, centrifuged for 2 min at 400 x g at 4°C, and fixed with 4% paraformaldehyde (Sigma‐Aldrich 158127) for 10 min at RT. Fixed cells were washed with PBS, permeabilized with perm buffer (Biolegend 421002), incubated with blocking buffer (5% BSA, 0.015% Triton in PBS) for 45 min and labeled with anti‐human active caspase‐3 antibody clone Asp175 (Cell Signaling Technologies 9661) used at 1:400. After 60 min incubation at 4°C, cells were washed with PBS and incubated with anti‐Rabbit IgG (H+ L) Alexa Fluor 546, (Invitrogen, A11010) used at 4 µg/mL (1:500) for 30 min at 4°C. Cells were washed with PBS and mounted in 5 µL of DAKO mounting medium, covered with coverslips, dried overnight and sealed with nail polish. Images were acquired on a Zeiss 880 Airyscan confocal microscope in superresolution mode and analyzed using ImageJ software.

### 4.3 Analysis of CD43 Shedding by ADAM10

CEM‐CD43_Halo_ cells were prepared as previously described [11]. Cells in logarithmic growth phase were washed twice and resuspended in fully supplemented RPMI medium (without antibiotics). All washing and centrifugation steps were performed at 1500 rpm for 1 min. Cells were labeled with 0.33 µm HaloTag Alexa Fluor 488 Ligand (Promega) to stain the CD43 ectodomain, gently mixed, and incubated at 37 °C for 1 h. After labeling, cells were washed twice and resuspended in 500 µL of supplemented, phenol red‐free L15 medium (Thermo Fisher). Cells were either left untreated, treated with 10 µm staurosporine at 37°C for 3 h, or pre‐treated with GI inhibitor (1:1000 dilution) at 37°C for 45 mins followed by 10 µm staurosporine for 3 h. The total incubation time was kept constant across all conditions. Cells were pelleted, and the supernatant was collected, centrifuged again, and transferred to a 0.5 mL 40K MWCO Zeba spin desalting column (Thermo Fisher), pre‐washed twice with PBS. The flowthrough was clarified twice more using 40K columns, and the final flowthrough was used for fluorescence correlation spectroscopy (FCS) measurements. Peaks were counted using FCS measurements which were performed using a Zeiss LSM 980 confocal microscope. Excitation of Alexa Fluor 488 was achieved using a 488 nm argon ion laser. A 40×/1.2 NA water immersion objective was used for focusing. For each sample, 10 fluorescence intensity curves (10 s each) were recorded. Laser power was set to 1% of the total output, corresponding to approximately 10 µW. Intensity traces were analyzed to count fluorescence peaks using single‐particle profiler software [30].

### 4.4 MD Simulations System Preparation

As a starting point for the simulations, we used the X‐ray structure of ADAM10 (PDB ID: 6BE6) [19]. Unbuilt residues (670–748) were reconstructed using Alphafold2 [41, 42], resulting in two juxtamembrane helices (654–677, StD), a single transmembrane helix (TmD) and a relatively unstructured IcD. Initial systems were built using CHARMM‐GUI [43] to embed the full‐length ADAM10 model in a lipid bilayer composed of either POPC or a mixture of 7:3 POPC:POPS. Each system was then solvated in a box of explicit water molecules and neutralized in 150 mm KCl. To ensure thorough sampling of the POPC‐POPS system, despite the short (100‐ns) timescales of individual MD trajectories relative to lipid diffusion, we generated 20 different initial membrane repartitions using the membrane mixer plugin in VMD (Movie S4). System compositions are described in Table 1.

**TABLE 1:** Composition of simulated systems.

System	Lipids	Water	Number of atoms	Duration	Dimensions (nm^3^)
POPC	761	150k	550k	32 µs	16 × 16 × 22
7:3 POPC:POPS	560:240	150k	550k	32 µs	16 × 16 × 22

MD simulations of ADAM10 were performed using GROMACS 2023.2 [44]. The CHARMM36m [45, 46] force field was employed in combination with the TIP3P [47] water model. The systems underwent energy minimization using the steepest descent algorithm until the energy gradient converged to a threshold of 0.01 kcal/mol/Å. The solvent and membrane were then allowed to relax in the NVT ensemble at 300K for 625 ps. Each system then underwent NPT equilibration for 3 ns, first increasing the timestep from 1 to 2 fs, then progressively removing all restraints on protein and lipids. The LINCS algorithm [48] was used for bond constraints.

During equilibration, the Berendsen thermostat [49] and barostat (when applicable) were used. For production, the v‐rescale thermostat [50] and c‐rescale barostat [51] were used for temperature and pressure control, respectively. For equilibration as well as production runs, a cutoff distance for non‐bonded interactions was set at 1.2 nm, utilizing a cutoff van der Waals type with a force‐switch modifier. The switching distance was configured to 1.0 nm. Coulombic interactions were calculated using the particle mesh Ewald (PME) method [52], with a cutoff distance of 1.2 nm.

### 4.5 Adaptive Sampling

We performed adaptive sampling based on the FAST method [27] to enhance exploration of the conformational landscape. Briefly, the method consists of setting up a swarm of short simulations, clustering structural models among the resulting trajectories, and selecting models among these to set up a new swarm with random initial velocities. The new starting models were chosen based on a reward function defined as the sum of 127 pairwise distances spread among 4 interfaces between the catalytic MpD and neighboring CrD and StD regions (Figure S1 and Table ST1). For each system we generated 16 generations of 20 simulations, each with a length of 50 ns. The first swarm of simulations started from a model based on the closed experimental structure with random initial velocities. For each new generation, the total accumulated simulations were considered in the clustering. Once the overall sampling was done, simulations from all seeds were extended to 100 ns, amounting to 32 µs of sampling for each system.

### 4.6 Statistical Analysis

To analyze flow cytometry data, ADAM10 expression was quantified by activated caspase‐3 labeling, normalized to the level in non‐apoptic cells. Data from 3 to 6 independent experiments are presented as individual values and mean % ADAM10 expression, with significance tested in Prism (GraphPad Software) using one‐way ANOVA of log10‐transformed data with Dunnett's post‐hoc test, ^****^ *p *< 0.0001, ns *p* > 0.9.

To analyze simulations data, MSMs were constructed using pyEmma [53]. To this end, we performed tICA [54] based on the 127 pairwise distances described above, along with pairwise distances between regions of ADAM10 interacting with Tspan15 in the open experimental structure (Figure S1 and Table ST2). As previously described [24, 55], faster dimensions represented by a single Gaussian distribution were discarded, and we focused subsequent analyses on the slowest two independent components. After VAMP‐2 validation [56], 200 micro‐states were clustered using *k*‐means [57] clustering (Figure S2). We used Bayesian Markov state modeling [58], with implied timescales over a range of lag times from 0.1 to 50 ns, to validate the use of a 10 ns lag time [59] (Figure S3). We then performed PCCA+ analysis [60] to build 4‐state coarse‐grained MSMs of both the POPC and POPC‐POPS systems. These latest models were validated using the Chapman‐Kolmogorov test [59], up to a lag time of 50 ns, corresponding to half the length of each individual simulation (Figure S4). Error analysis regarding populations and MFPTs was performed following a Bayesian scheme using *n* = 100 samples (Table ST3). Relevant observables were computed using VMD [61], and results were displayed using seaborn [62] and matplotlib [63].

## Author Contributions

A.S. led the investigation, formal analysis, methodology, resources, software, validation, visualization, and writing of the original draft. N.H. contributed to the investigation, methodology, resources, and software. M.C. contributed to the conceptualization, funding acquisition, methodology, project administration, supervision, reviewing, and editing the draft. S.Z. contributed to the investigation, methodology, resources, and validation. Q.J.S. contributed to conceptualization, formal analysis, investigation, methodology, resources, supervision, reviewing, and editing the draft. E.S. contributed to formal analysis, funding acquisition, methodology, project administration, resources, supervision, and reviewing and editing the draft. L.D. led the funding acquisition and project administration, and contributed to conceptualization, resources, supervision, and reviewing and editing the draft. R.J.H. led the project administration, resources, supervision, and contributed to conceptualization, funding acquisition, investigation, and reviewing and editing the draft.

## Conflicts of Interest

The authors declare no conflicts of interest.

## Supporting information




**Supporting File 1**: advs74049‐sup‐0001‐SuppMat.pdf.

**Supporting File 2**: advs74049‐sup‐0002‐MovieS1.mp4.

**Supporting File 3**: advs74049‐sup‐0003‐MovieS2.mp4.

**Supporting File 4**: advs74049‐sup‐0004‐MovieS3.mp4.

**Supporting File 5**: advs74049‐sup‐0005‐MovieS4.mp4.

## Data Availability

Input files and trajectories for MD simulations in POPC are available at https://doi.org/10.5281/zenodo.15863909, and in mixed POPC:POPS at https://doi.org/10.5281/zenodo.15864196.
