# Supplementary material for: Lipid‐Facilitated Opening of the ADAM10 Sheddase Revealed by Enhanced Sampling Simulations
Source: Adv Sci (Weinh). 2026 Feb 24;13(19):e15713. doi: 10.1002/advs.202515713 (PMC13045381; doi:10.1002/advs.202515713)
Supplement: Supplementary file 1 — Supporting File 1: advs74049‐sup‐0001‐SuppMat.pdf. [file ADVS-13-e15713-s003.pdf]

Supplementary Information:

Lipid-facilitated opening of the ADAM10 sheddase revealed by enhanced sampling simulations

Adrien Schahl[1,*], Nandan Haloi[1], Marta Carroni[2], Shengpan Zhang[3,4], Quentin James Sattentau[4,5], Erdinc Sezgin[6,*] , Lucie Delemotte[1,*], Rebecca J Howard[2,*]

[1]SciLifeLab, Department of Applied Physics, KTH Royal Institute of Technology, 17121 Solna, Sweden

[2]SciLifeLab, Department of Biochemistry and Biophysics, Stockholm University, 17121 Solna, Sweden

[3]The Kennedy Institute of Rheumatology, University of Oxford, Roosevelt Drive, Oxford OX3 7FY, UK

[4]Sir William Dunn School of Pathology, University of Oxford, Oxford OX1 3RE, UK

[5]The Max Delbrück Centre for Molecular Medicine, Campus Berlin-Buch, 13125 Berlin, Germany

[6]SciLifeLab, Department of Women's and Children's Health, Karolinska Institutet, 17121 Solna, Sweden

* Correspondence:

adrien.schahl@scilifelab.se

erdinc.sezgin@ki.se

lucie.delemotte@scilifelab.se

rebecca.howard@scilifelab.se

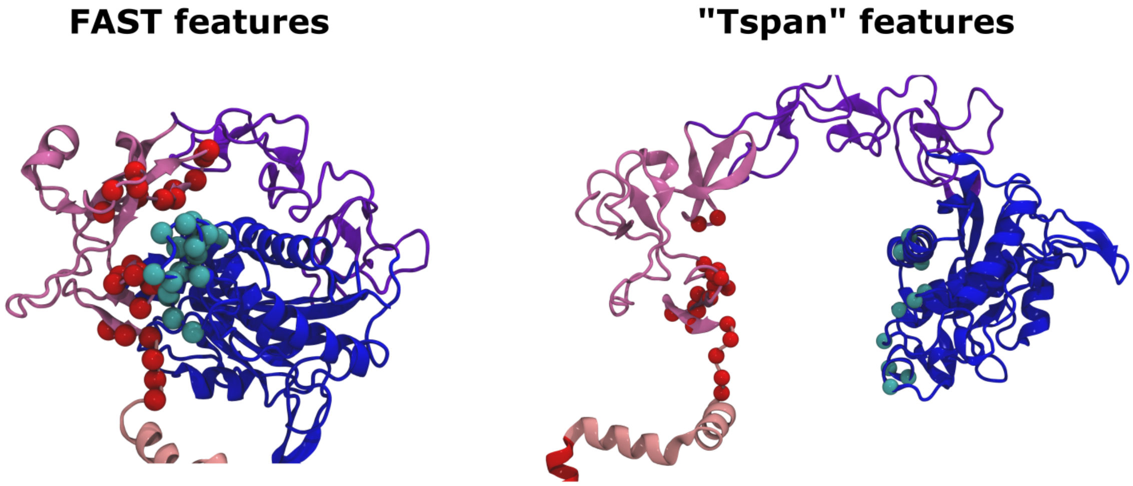

Supplementary Figure S1. Features used for adaptive sampling and MSM building.
Left: Residues of the MpD (cyan spheres) and CrD/StD regions (red spheres) used to define interdomain distances to select seeds for successive generations of FAST sampling.
Right: Additional residues of the MpD (cyan spheres) and CrD/StD regions (red spheres) used to define additional features for the construction of MSMs based on FAST sampling. Specific residue numbers are listed in Supp. Tab. ST1 and ST2.

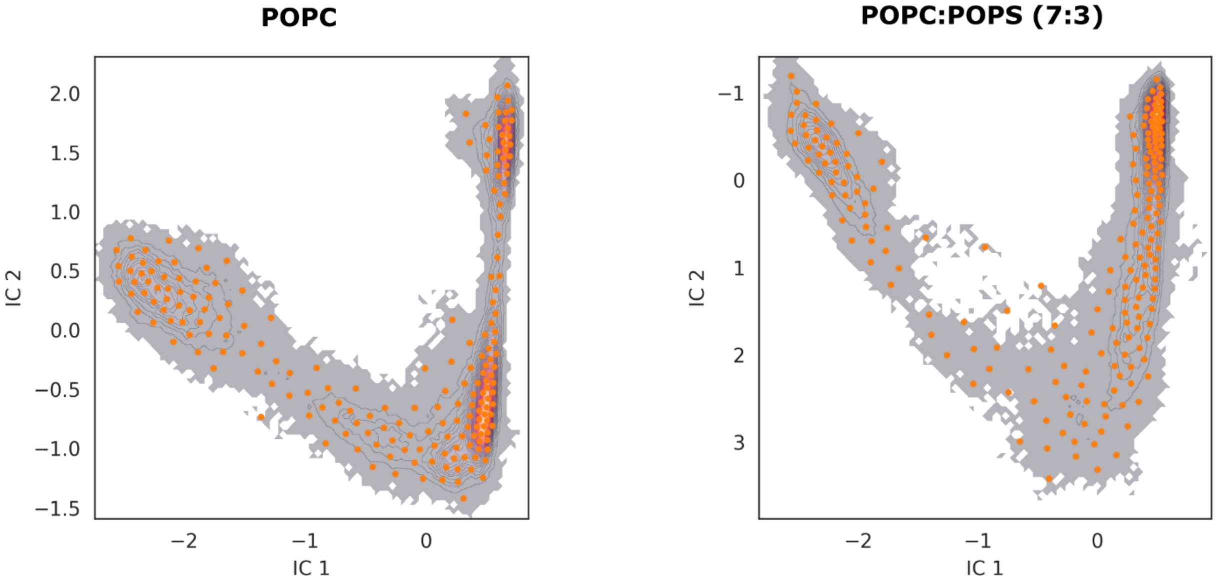

Supplementary Figure S2. Distribution of centroids (orange) obtained by *k*-means clustering in tICA space. 200 microstates were assigned in both POPC-only (left) and POPC-POPS (right) conditions.

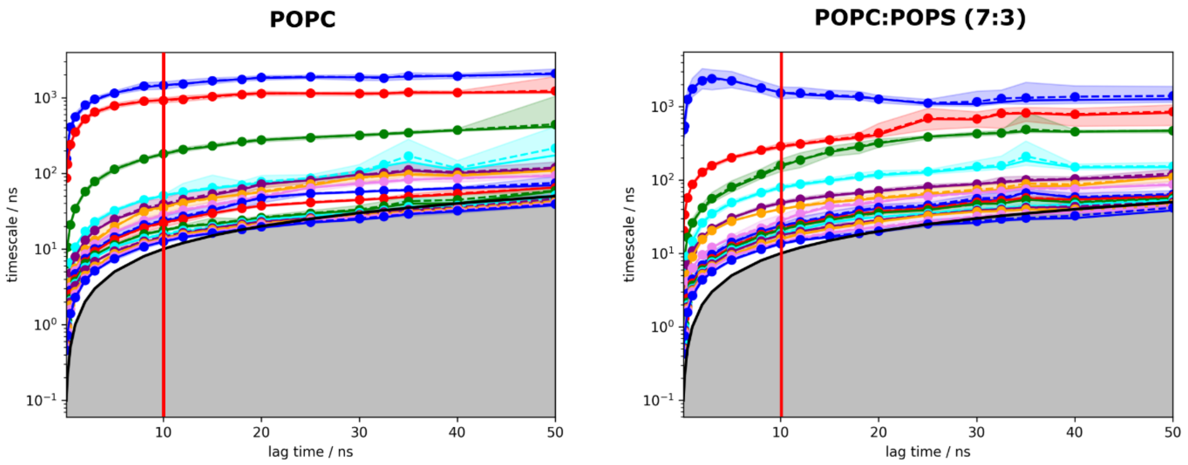

Supplementary Figure S3. Assessment of convergence of MSMs with implied timescales. Colored series represent the first 10 implied timescales representing the 10 slowest processes of the MSM, for POPC-only (left) and POPC-POPS (right) systems. Vertical red line represents the MSM lag time selected for each system, chosen as the smallest lag time where the implied timescales have leveled out.

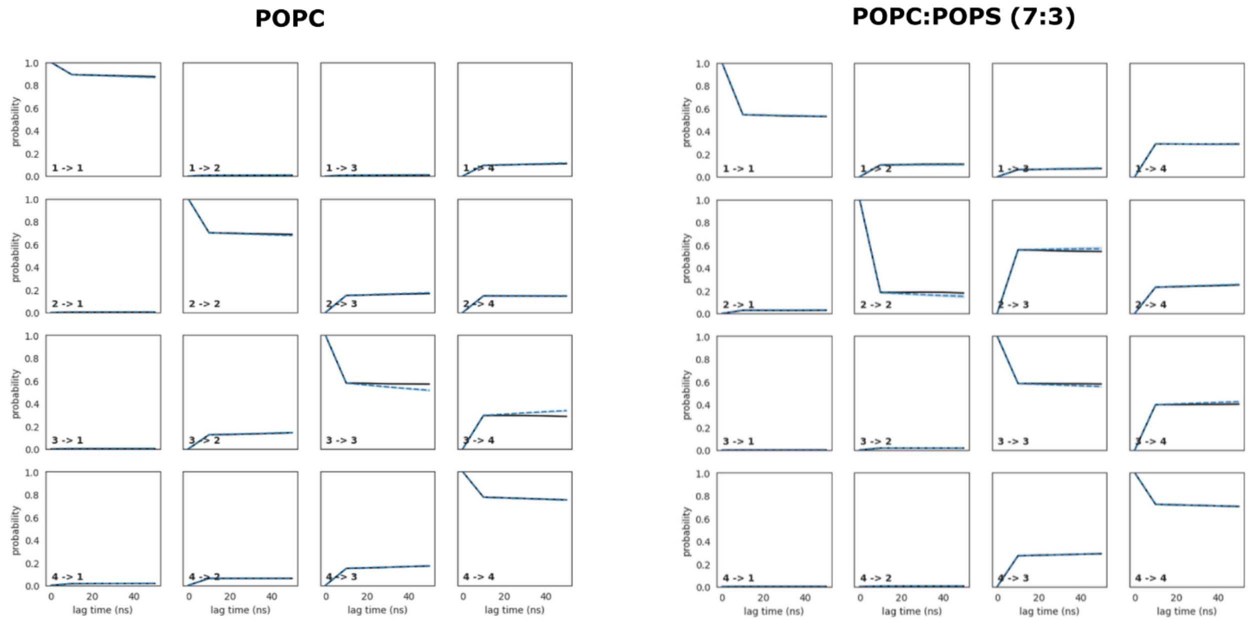

Supplementary Figure S4. Assessment of convergence of 4-state MSMs in POPC and 7:3 POPC:POPS systems using Chapman-Kolmogorov tests.

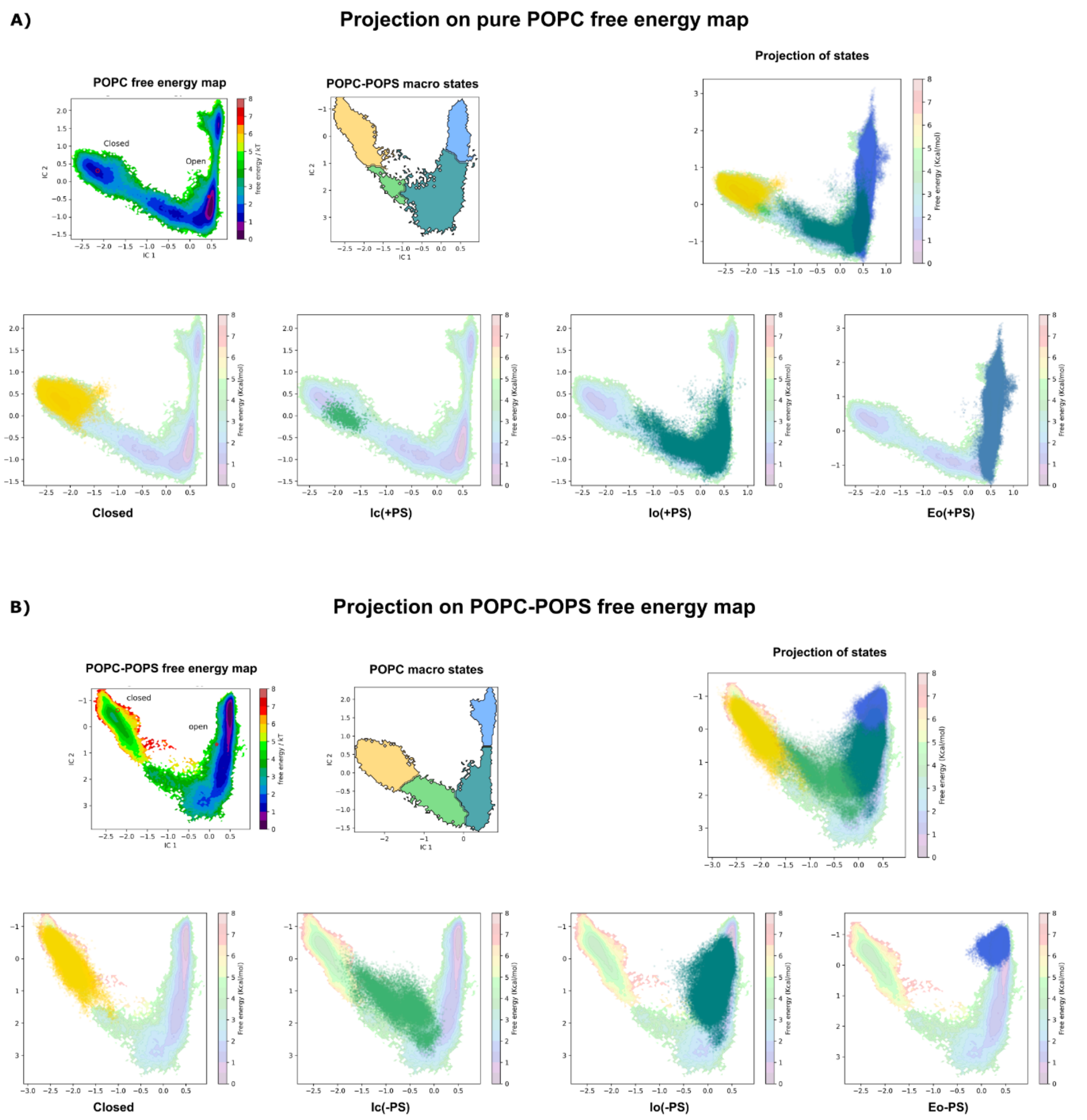

Supplementary Figure S5. Comparison of coarse-grained macro-states in the absence and presence of PS. A) *Bottom,* projection of simulation frames corresponding to (left to right) Closed (yellow), Ic(+PS) (green), Io(+PS) (teal) and Eo(+PS) (blue) macro-states from the POPC-POPS system onto the free-energy map computed in pure POPC, colored semi-transparent according to scalebar at right. For reference, *top* shows (left-to-right) the free-energy landscape for the POPC system without transparency (reproduced from Fig. 3C), coarse-grained MSM for the POPC-POPS system (reproduced from Fig. 4C) and projections of all four macro-states in POPC-POPS onto the free-energy landscape in POPC.
B) *Bottom,* projection of simulation frames corresponding to (left to right) Closed (yellow), Ic(-PS) (green), Io(-PS) (teal) and Eo(-PS) (blue) macro-states from the pure-POPC system onto the free-energy map computed in POPC-POPS, colored semi-transparent according to scalebar at right. For reference, *top* shows (left-to-right) the free-energy landscape for the POPC-POPS system without transparency (reproduced from Fig. 3E), coarse-grained MSM for the POPC-only system (reproduced from Fig. 4A) and projections of all four macro-states in POPC alone onto the free-energy landscape in POPC-POPS.

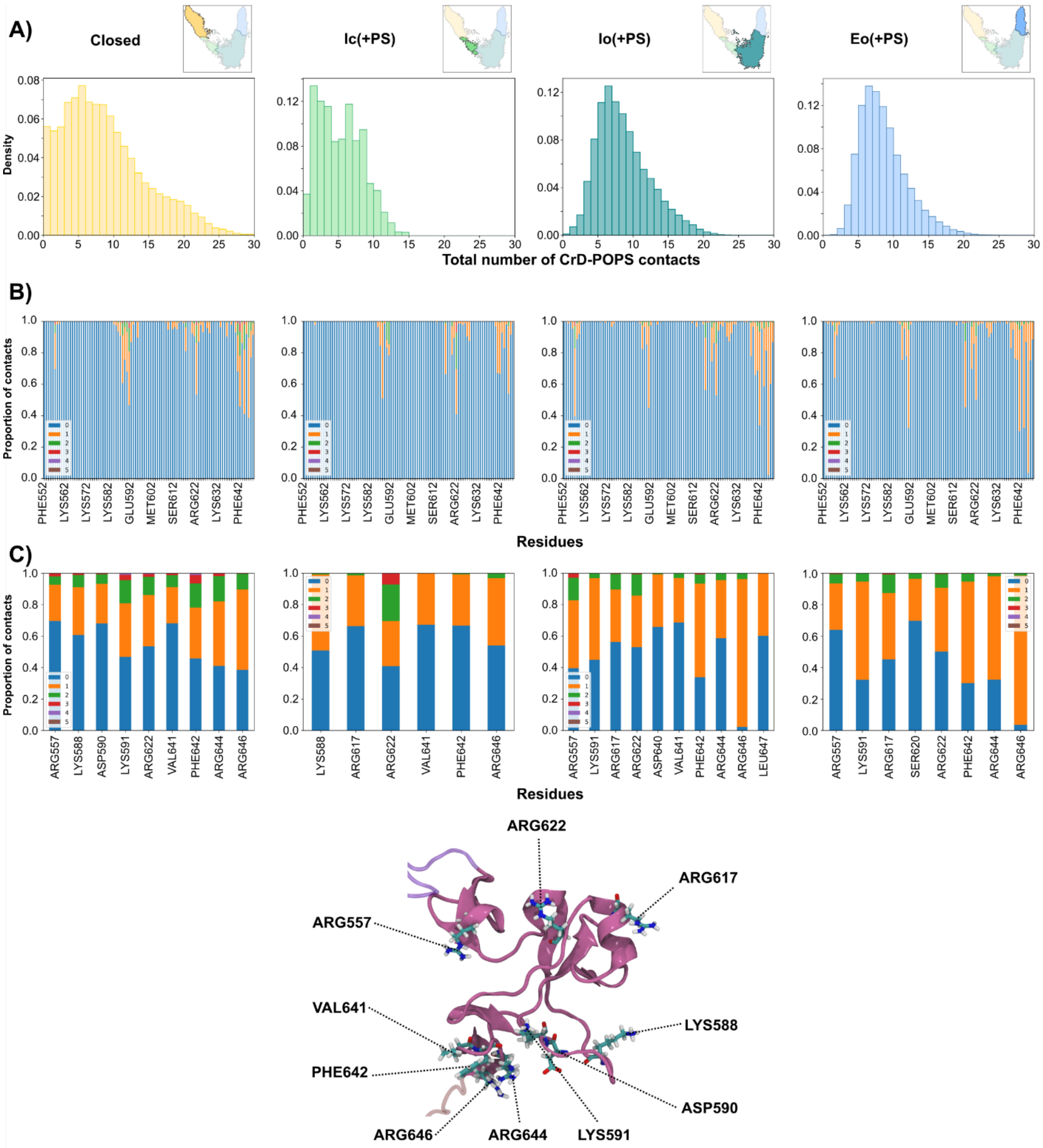

Supplementary Figure S6. PS interactions with basic residues in the ADAM10 CrD.
A) Histograms of the number of PS contacts with the CrD in simulation frames corresponding to the (left to right) Closed (yellow), Ic(+PS) (green), Io(+PS) teal or Eo(+PS) macro-states obtained in POPC-POPS conditions. *Insets* represent projections of the corresponding macro-state in tICA space, as originally shown in Fig. 4C. **B)** Proportion of simulation frames in which a given residue in the CrD makes 0 (blue), 1 (orange), 2 (green) etc. contacts with PS lipids in each macro-state defined in panel A. **C)** Contact proportions as in panel B for residues making at least one PS contact in >30% of simulation frames in a given macro-state. Inset below shows positioning of frequent PS contacts (cyan sticks) in a cartoon representation of the CrD (magenta). ´

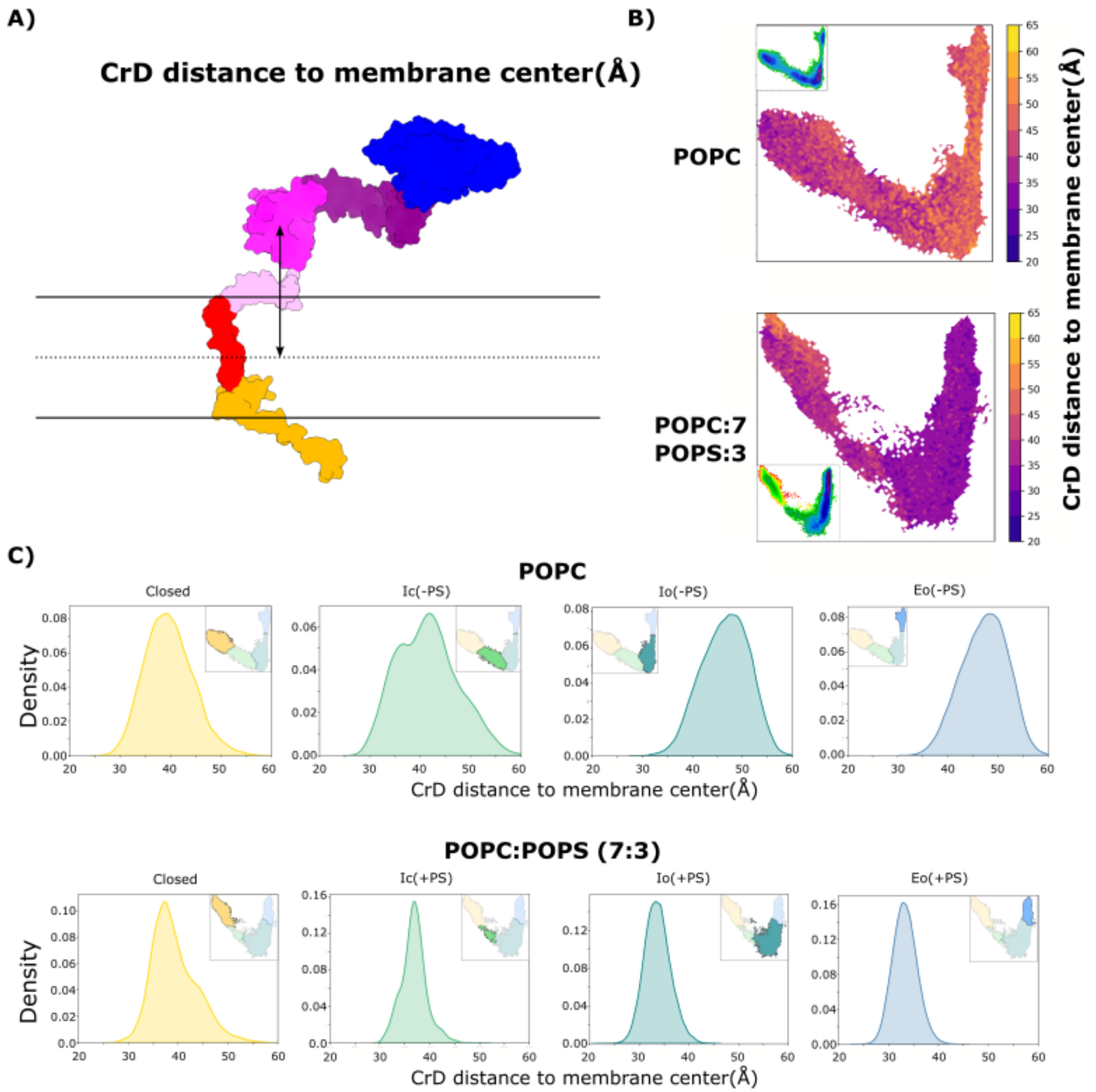

Supplementary Figure S7. The CrD is retained proximal to membrane lipids in the presence of PS. A) Schematic showing the distance (arrow) between the CrD (magenta) and the center of the membrane (dashed line). **B)** CrD-membrane distances, measured as in panel A and colored according to righthand scalebar, projected onto tICA maps based on POPC (above) and POPC-POPS (below) systems. For reference, insets reproduce free-energy landscapes from Fig. 3. **C)** Probability distributions of CrD-membrane distances for the indicated macro-states obtained in POPC (above) and POPC-POPS (below) conditions. Insets represent projections of the corresponding macro-state in tICA space, as originally shown in Fig. 4.

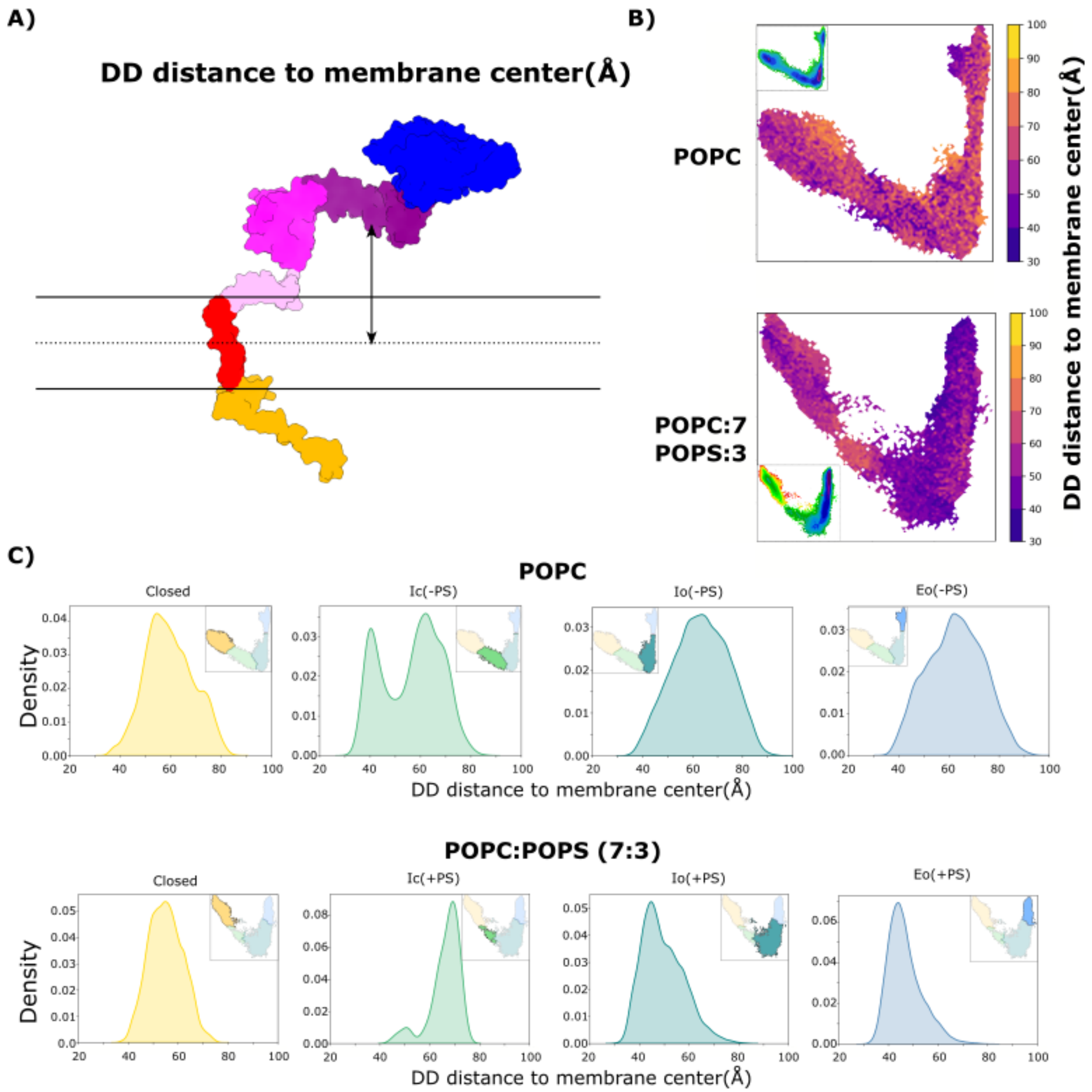

Supplementary Figure S8. The DD is retained proximal to membrane lipids in the presence of PS. A) Representation of the distance between the DD and the center of the membrane. For comparison, in-sets represent the free energy maps of Figure 3. **B)** Projection of this distance over free energy maps in POPC and POPC-POPS simulations. **C)** Distribution of this distance in each macro state obtained in both conditions. In-sets represent the Macro-state projection of Figure 4.

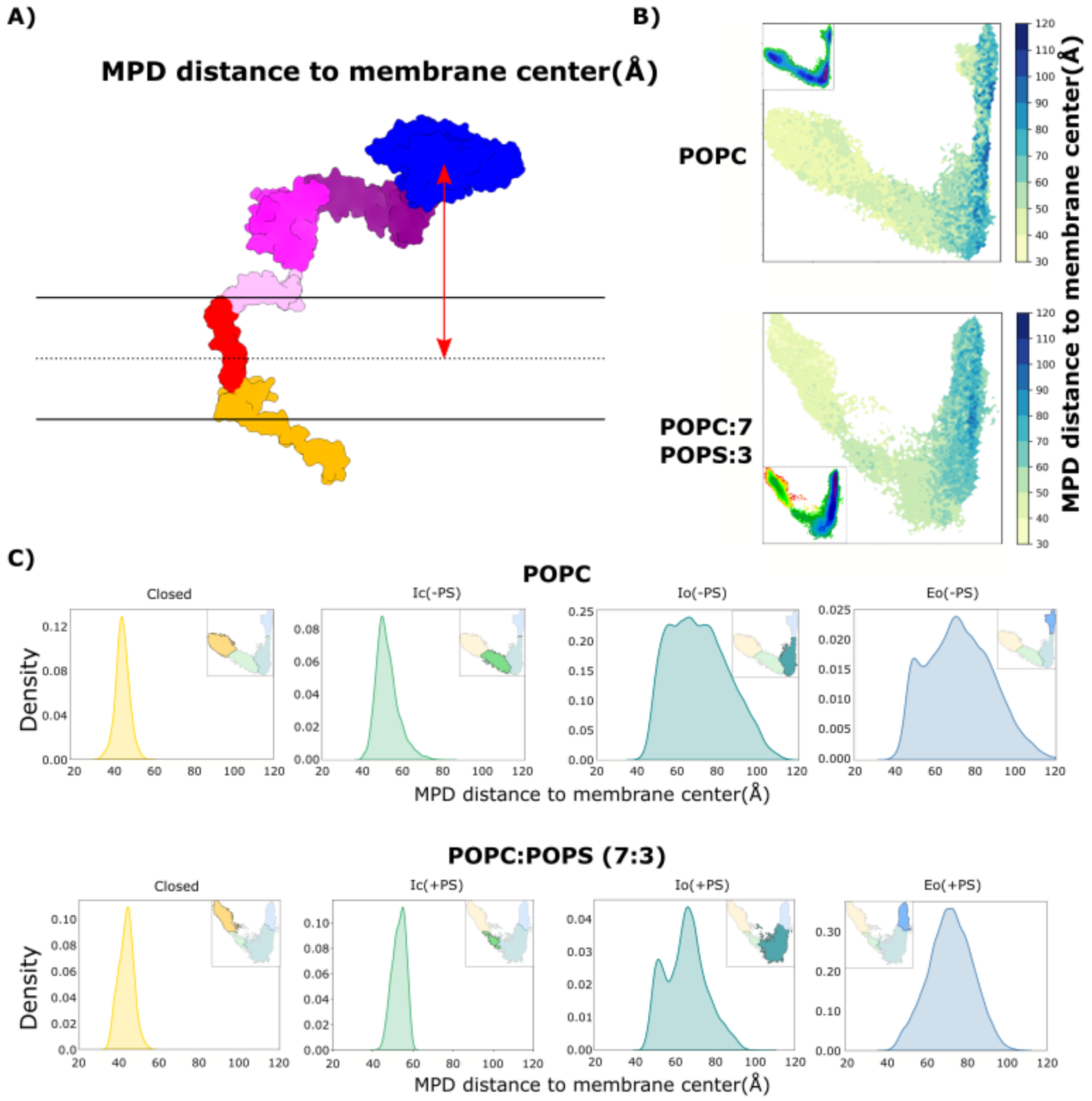

Supplementary Figure S9. MpD dissociates from membrane lipids early in ADAM10 opening in the absence or presence of PS. A) Schematic showing the distance (arrow) between the MpD (blue) and the center of the membrane (dashed line). **B)** MpD-membrane distances, measured as in panel A and colored according to righthand scalebar, projected onto tICA maps based on POPC (above) and POPC-POPS (below) systems. For reference, insets reproduce free-energy landscapes from Fig. 3. **C)** Probability distributions of MpD-membrane distances for the indicated macro-states obtained in POPC (above) and POPC-POPS (below) conditions. Insets represent projections of the corresponding macro-state in tICA space, as originally shown in Fig. 4.

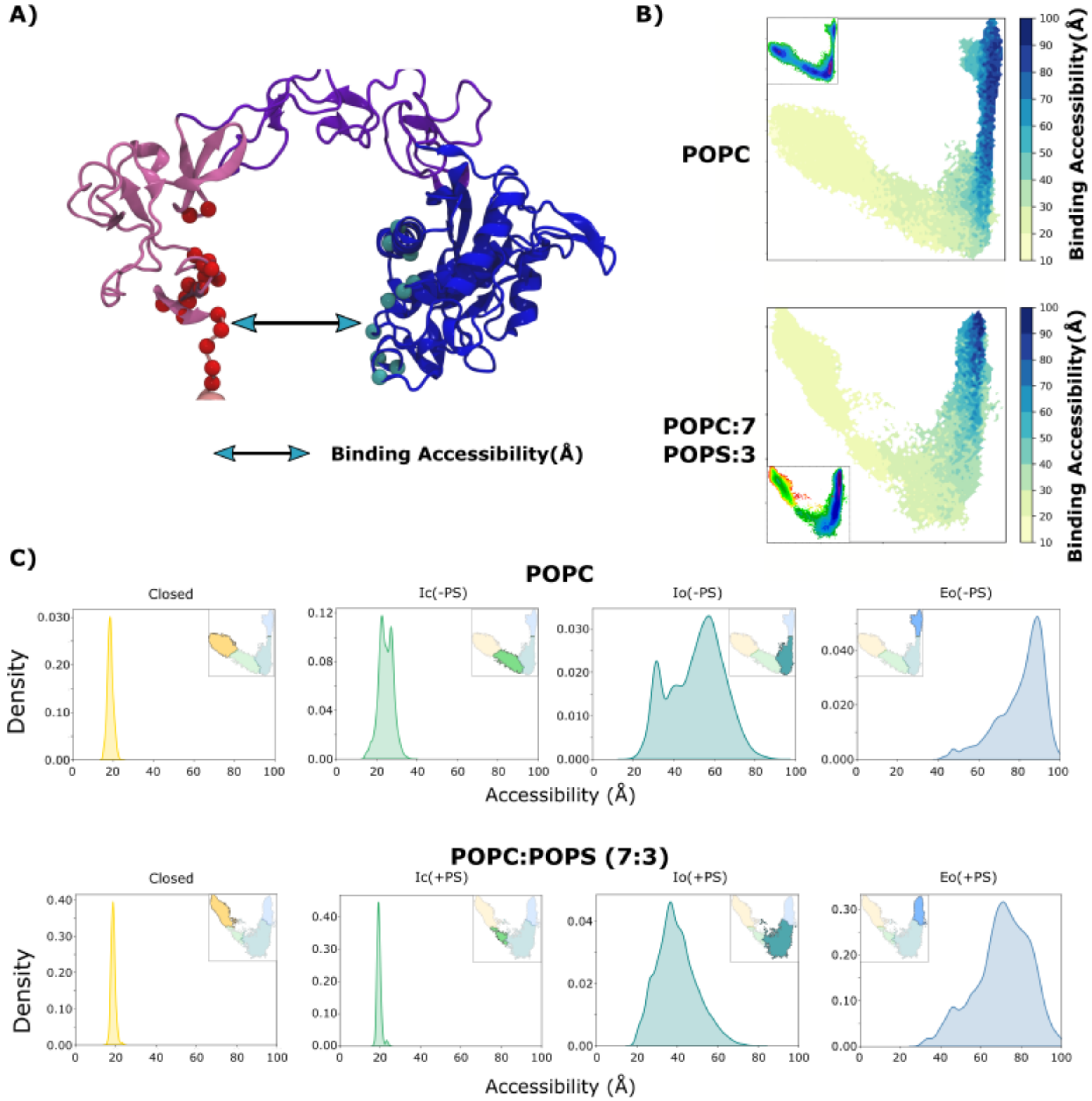

Supplementary Figure S10. Structural accessibility of ADAM10 to Tspan binding.
A) Schematic of pairwise interdomain distances (arrow) between residues in the MpD (cyan) and CrD/StD regions (red) defining the binding cleft for Tspan15. **B)** Summed pairwise distances representing Tspan accessibility, measured as in panel A and colored according to righthand scalebar, projected onto tICA maps based on POPC (above) and POPC-POPS (below) systems. For reference, insets reproduce free-energy landscapes from Fig. 3.
C) Probability distributions of Tspan accessibility for the indicated macro-states obtained in POPC (above) and POPC-POPS (below) conditions. Insets represent projections of the corresponding macro-state in tICA space, as originally shown in Fig. 4.

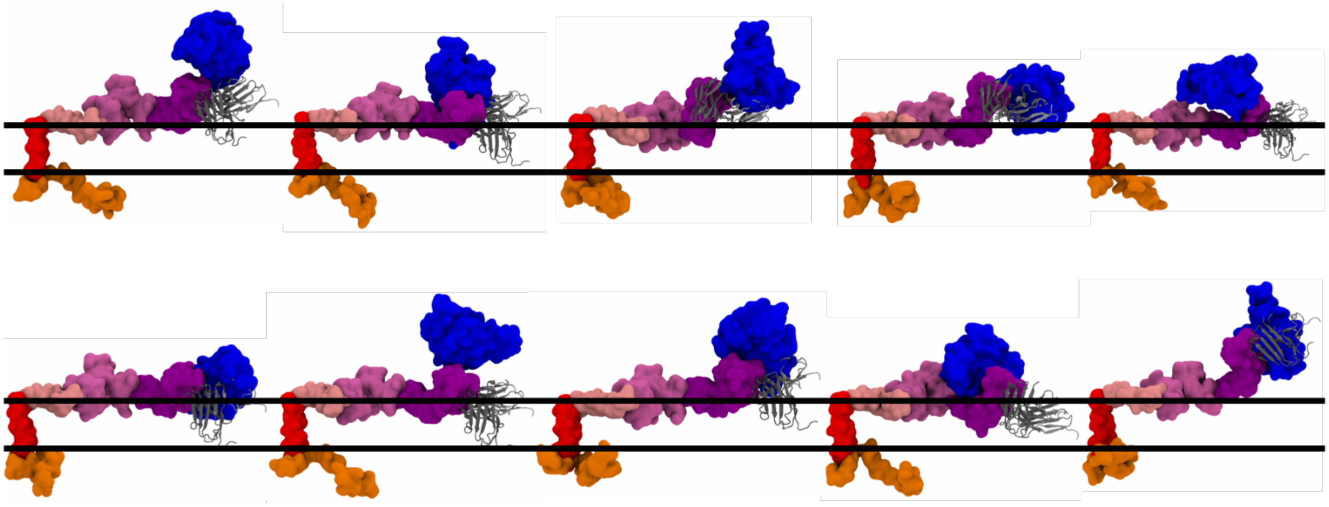

Supplementary Figure S11. Predicted Fab-fragment binding to ADAM10 in representative snapshots from the most represented state in POPC-POPS conditions. Ten frames were randomly extracted from the most populated state (Eo(+PS)) in the POPC-POPS system using the "sample_by_distributions" routine in pyEmma. The Fab_{cryo} structure (PDB ID: 8ESV, gray ribbons) was then superimposed onto each snapshot based on the ADAM10 DD (purple surface). For reference, the membrane periphery observed in MD simulations is represented by black lines.

Supplementary Table ST1. Residues used to define pairwise interdomain distances for FAST adaptive sampling.

MPD	CrD/StD	MPD	CrD/StD	MPD	CrD/StD
374	564	408	602	413	633
375	564	409	602	414	633
376	564	410	602	401	634
424	564	411	602	402	634
425	564	412	602	403	634
426	564	407	603	404	634
374	565	408	603	405	634
375	565	409	603	413	634
376	565	410	603	414	634
424	565	411	603	401	635
425	565	412	603	402	635
426	565	407	604	403	635
374	567	408	604	404	635
375	567	409	604	405	635
376	567	410	604	413	635
424	567	411	604	414	635
425	567	412	604	401	636
426	567	401	630	402	636
374	568	402	630	403	636
375	568	403	630	404	636
376	568	404	630	405	636
424	568	405	630	413	636
425	568	413	630	414	636
426	568	414	630	419	646
374	569	401	631	420	646
375	569	402	631	421	646
376	569	403	631	419	647
424	569	404	631	420	647
425	569	405	631	421	647
426	569	413	631	419	648
374	570	414	631	420	648
375	570	401	632	421	648
376	570	402	632	419	649
424	570	403	632	420	649
425	570	404	632	421	649
426	570	405	632	419	650
407	601	413	632	420	650
408	601	414	632	421	650
409	601	401	633	419	651
410	601	402	633	420	651
411	601	403	633	421	651
412	601	404	633		
407	602	405	633		

Supplementary Table ST2. Residues used to define pairwise interdomain distances for Tspan accessibility. Distances in this list were combined with those in Supp. Tab. ST1 as features to build coarse-grained MSMs.

MPD	CrD/StD
248	556
249	556
251	556
248	557
249	557
251	557
375	627
378	627
375	628
378	628
375	629
378	629
375	630
378	630
375	631
378	631
375	635
378	635
407	638
410	638
411	638
407	639
410	639
411	639
407	640
410	640
411	640
407	641
410	641
411	641
407	642
410	642
411	642
407	646
410	646
411	646
425	648
430	648
425	649
430	649
425	650
430	650
425	651
430	651
425	652
430	652

Supplementary Table ST3. Values and errors evaluated from the Bayesian posterior distribution for MSM transitions. *Above,* populations in each of 4 states in POPC-only (left) and POPC-POPS (right) systems. *Below,* MFPTs for transitions between each possible pair among 4 states in POPC-only (left) and POPC-POPS (right) systems. Each evaluation uses n = 100 samples.

States (POPC)

	Value(%)	error(%)
Closed	19.9	±0.5
Ic(-PS)	19.5	±0.6
Io(-PS)	53.2	±0.6
Eo(-PS)	7.3	±0.3

States (POPC-POPS)

	Value(%)	error(%)
Closed	0.4	±0.01
Ic(+PS)	0.3	±0.1
Io(+PS)	40.9	±0.6
Eo(+PS)	58.4	±0.7

MFPTs (POPC)

From	to	Value(μs)	error(μs)
Closed	Ic(-PS)	0.5	±0.3
	Io(-PS)	1.6	±0.09
	Eo(-PS)	16.9	±1.8
Ic(-PS)	Closed	2.9	±1.8
	Io(-PS)	0.5	±0.05
	Eo(-PS)	15.8	±1.8
Io(-PS)	Closed	4.3	±0.3
	Ic(-PS)	0.8	±0.03
	Eo(-PS)	14.9	±1.3
Eo(-PS)	Closed	5.8	±0.3
	Ic(-PS)	2.3	±0.1
	Io(-PS)	1.1	±0.07

MFPTs (POPC-POPS)

From	to	Value(μs)	error(μs)
Closed	Ic(+PS)	4.9	±1.1
	Io(+PS)	1.5	±0.1
	Eo(+PS)	2.2	±0.1
Ic(-PS)	Closed	270	±54
	Io(+PS)	0.1	±0.1
	Eo(+PS)	0.8	±0.03
Io(-PS)	Closed	300	±55
	Ic(+PS)	34	±54
	Eo(+PS)	0.3	±0.01
Eo(-PS)	Closed	300	±55
	Ic(+PS)	36	±6
	Io(+PS)	0.4	±0.01

Supplementary Video 1. Animation showing final frames from 320 progressively seeded FAST-sampling trajectories in a POPC-only bilayer, revealing opening of ADAM10, while retaining the general fold within each domain.

Supplementary Video 2. Animation showing final frames from 320 progressively seeded FAST-sampling trajectories in a POPC-POPS bilayer, revealing opening of ADAM10, while retaining the general fold within each domain.

Supplementary Video 3. Animation showing 5 models of the ADAM10-Fab$_{fluo}$ complex generated in AlphaFold3, viewed in two orientations, showing consistent contacts of the ADAM10 DD (magenta ribbons) with the Fab$_{fluo}$ heavy and light chains (yellow and green ribbons, respectively). The ADAM10 MpD (blue) and CrD (pink) are shown as opaque surfaces.

Supplementary Video 4. Animation showing 20 distinct initial membrane repartitions generated using the membrane mixer plugin in VMD to seed FAST-sampling simulations in 7:3 POPC:POPS bilayers.
